# Neurobrucellosis Presenting with Motor Damage or Hearing Loss, and Use of Steroids are Associated with a Higher Risk of Sequelae or Relapse: A Systematic Review of Individual Participant Data

**DOI:** 10.1007/s10072-024-07621-6

**Published:** 2024-06-11

**Authors:** Chiara Fusetti, Francesco Petri, Mohammad H. Murad, Stefania Merli, Riccardo Giorgi, Giuliano Rizzardini, Andrea Gori, Matteo Passerini

**Affiliations:** 1 Department of Infectious Diseases, ASST Fatebenefratelli Sacco, “L. Sacco” University Hospital, Via Giovanni Battista Grassi N. 74, 20157 Milan, Italy; 2 Department of Medicine, Mayo Clinic, Division of Public Health, Infectious Diseases and Occupational Medicine, 200 1St St SW, Rochester, MN 55905 USA; 3 Evidence-Based Practice Center, Mayo Clinic, 200 1St St SW, Rochester, MN 55905 USA; 4 Department of Pathophysiology and Transplantation, University of Milan, Via Francesco Sforza N. 35, 20122 Milan, Italy; 5 Centre for Multidisciplinary Research in Health Science (MACH), University of Milan, Via Francesco Sforza N. 35, 20122 Milan, Italy

**Keywords:** Brucellosis, Motor impairment, Hearing loss, Steroids, Duration of therapy

## Abstract

**Background:**

Neurobrucellosis presents diverse clinical challenges and risks of long-term complications.

**Objective:**

We aimed to assess the relationship between the duration of antibiotic therapy, clinical factors, and the outcome of neurobrucellosis with a case report combined with a systematic review of the literature.

**Methods:**

We present a case of a 31 years-old man successfully treated at our Institution. We then searched Ovid MEDLINE, Embase and Scopus for articles that encompassed neurobrucellosis cases, duration of treatment, and outcome. The primary outcome was to assess an association between the duration of treatment and the risk of sequelae or relapses. Univariate, multivariate and sensitivity analysis were carried out to define which variables affect​ed​ the clinical outcome. Quality assessment was performed using a dedicated tool.

**Results:**

A total of 123 studies were included, totaling 221 patients. Median duration of treatment was 4 months (IQR 3 – 6), 69% patients recovered without sequelae, 27% had sequelae. Additionally, five patients had a relapse, and 4 patients died. Multivariate analysis found that the duration of treatment, age, and the use of ceftriaxone were not associated with a higher risk of sequelae or relapses. A significant association was found for corticosteroids use (OR 0.39, 95% IC 0.16 – 0.96, p = 0.038), motor impairment (OR 0.29, 95% IC 0.14 – 0.62, p = 0.002), and hearing loss (OR 0.037, 95% IC 0.01 – 0.11, p < 0.001).

**Conclusions:**

This study highlights the variability in clinical presentations and treatment approaches for neurobrucellosis. Patients with factors indicating higher sequelae risk require meticulous follow-up.

**Supplementary Information:**

The online version contains supplementary material available at 10.1007/s10072-024-07621-6.

## Introduction

Brucellosis is a zoonotic infection transmitted to humans from infected animals by ingestion of unpasteurized dairy products or by contact or inhalation of tissue or fluids [1, 2].

Brucellosis is endemic in the Mediterranean basin, Middle East, Central Asia, China, the Indian subcontinent, Sub-Saharan Africa, Central, and South America. The cases of brucellosis are sporadic outside these areas, mostly imported and favored by migratory flows or tourism [2].

Neurobrucellosis complicated less than 5% of brucellosis cases. The main manifestations include meningitis, meningoencephalitis, myelitis, radiculitis, peripheral neuropathies, cerebrovascular involvement, and psychiatric manifestations. Combined antibiotic therapy allows to reduce the risk of therapeutic failure and relapse [2, 3].

The duration of treatment is not clearly identified, with some authors suggesting treating neurobrucellosis until the normalization of the cerebrospinal fluid (CSF) parameters. Instead, in other cases the duration of the treatment is predetermined [2, 3].

The following case report combined with a systematic review of the literature and a pooled analysis of individual data aims to evaluate the possible correlation between the duration of antibiotic therapy and the clinical outcome, and subsequently to study how other factors could influence the outcome of patients, to provide tools to guide treatment in an uncommon pathology in Western countries and on which defined therapeutic indications are lacking.

## Methods

### Case report

We reported the most relevant information of a patient admitted to our hospital with neurobrucellosis. We collected data regarding the patient’s demographic characteristics, signs and symptoms, radiological, and microbiological findings from the first access to the emergency department and during the follow-up. Information on antimicrobial therapy and its duration has also been reported.

### Systematic review and pooled analysis

#### Data sources and search strategies

A medical reference librarian performed a systematic search of Ovid MEDLINE 1946 + , Embase 1974 + and Scopus, starting from the search terms “neurobrucellosis” AND “treatment” with the inputs of the investigators up to 8th September 2022. Articles not in English were excluded. Specific search terms and syntax are available in the Supplementary (Table S1).

This systematic review was performed in compliance with the Preferred Reporting Items for Systematic review and Meta-Analysis (PRISMA) guidelines [4] (Table S5). Furthermore, the details of this systematic review have been registered a priori on the PROSPERO site (No. CRD 42023405076).

#### Study selection, definitions, and quality assessment

We included the studies with sufficient details to define the duration of antibiotic therapy and the clinical outcome. The following parameters were used to define the clinical cases of neurobrucellosis: (i) clinical presentation, radiological imaging, and/or CSF alterations compatible with central or peripheral nervous system infection [2] AND (ii) culture, serology (standard tube agglutination test ≥ 1:160 on blood, by any title on CSF) and/or Polimerase Chain Reaction (PCR) positive on CSF, other central nervous system (CNS) material, blood and/or bone marrow. Clinical outcome was reported as “recovered”, “recovered with sequalae”, “relapse”, or “death”.

Two reviewers (C.F. and F.P.) screened all titles and abstracts independently. Studied included were included for the full-text screening performed by the same reviewer pair. Conflict resolution was carried out through a third reviewer (M.P.).

Quality assessment was performed using a proper tool to assess the methodological quality of case series and case reports [5].

#### Data extraction and type of outcome measure

For each included paper we collected the name of the first author, the year of publication, and the study design. Then, we extracted from each paper the individual data of the patients described, when possible. The data collected were:

(i) demographic characteristics: age and gender;
(ii) symptoms: fever, headache, nausea or vomiting, muscular weakness, hearing impairment, back pain, joint pain, sensitive alterations, sweating, psychiatric symptoms, urinary retention or incontinence, malaise or fatigue, dizziness, myalgia, chills, fecal incontinence, and other symptoms;
(iii) signs of the patients: decreased muscular strength, decreased or increased deep tendon reflex, meningeal irritation signs, confusion, hepatosplenomegaly, hypoesthesia and/or paresthesia, convulsions, hemiparesis, paraplegia, dysarthria, aphasia, diplopia, papilledema, ataxia, positive Babinski sign, ascites, sensorineural hearing loss, paraparesis, tremor, nystagmus, positive Romberg’s test and decreased visual acuity;
(iv) onset of symptoms: hearing loss in months, deterioration of vision in days, psychiatric symptoms in months, neurological symptoms in days and general symptoms in months;
(v) previous brucellosis;
(vi) microbiological findings: culture, serology and/or PCR positive for *Brucella spp.*;
(vii) CSF findings: abnormal opening pressure, increased white blood cell and/or lymphocytes, decreased glucose level, increased protein level;
(viii) radiological findings: no alterations, leptomeningeal, basal meningeal, spinal root and/or cranial nerve enhancement, abscess, granuloma, arachnoiditis, periventricular and/or deep white matter hyperintensity, leukoencephalopathy, demyelinating plaque, vascular involvement, hydrocephalus, and edema;
(ix) clinical manifestation: only peripherical, Guillain-Barrè syndrome, cranial nerves involvement, upper motor neuron involvement, increased intracranial pressure, radiculopathy, CNS abscess, hydrocephalus, stroke, peripheral neuropathy, CNS granulomas, ventriculoperitoneal shunt infection, myelitis, sinus thrombosis, syndrome of inappropriate anti-diuretic hormone secretion, diabetes insipidus, subdural empyema, mycotic aneurysm, arachnoiditis, vasculitis, meningitis, meningoencephalitis;
(x) treatment: duration of antibiotic treatment in months, use of ceftriaxone and/or corticosteroids, post-treatment follow-up and duration in months, lumbar puncture repetition at the end of treatment;
(xi) outcome: recovered, recovered with sequelae (sensorineural hearing loss, vision impairment, motor impairment, cognitive dysfunction, other sequalae), relapse, and death.
(xii) The primary outcome was the association between duration of treatment and clinical outcome. Secondly, we assessed the role of other factors (age, use of ceftriaxone, use of corticosteroids, motor impairment, hearing loss, and duration of symptoms before hospital admission) on the clinical outcome.

#### Summary of statistical analysis, software used, and certainty of evidence

Continuous variables were expressed as media and median, according to their distribution, while categorical variables were expressed in percentages for descriptive analysis. We performed an univariate analysis assessing the role of age, duration of the antibiotic treatment, expressed both as continuous and as categorical variables (treatment duration ≥ 6 months or less), use of ceftriaxone, use of corticosteroids, motor impairment, hearing loss, and pediatric age (< 18 years old), on the clinical outcome (recovered *versus* recovered with sequelae or relapse).

A multivariate analysis was carried out to define which variables affected the clinical outcome. Variables a priori included in the analysis were: duration of the antibiotic treatment, use of corticosteroids to treat the episode (more than one dose), sensorineural hearing loss, and motor impairment.

The motor impairment was defined as the presence of one or more of the following signs or symptoms: decreased muscular strength, hemiparesis, paraplegia, paraparesis, ataxia.

A second multivariate analysis was performed adding the duration of symptoms before hospital admission.

Finally, a sensitivity analysis was performed excluding pediatric patients from the univariate and multivariate analyses. Data were analyzed using R Statistical Software (v4.1.2; R Core Team 2021).

Certainty of evidence (CoE) was assessed using the GRADE approach [6].

## Results

### Case report

A 31-year-old male with an unremarkable past medical history, presented to our emergency department in May 2021 for two-week history of difficulty in speech and deflection of mood. Hyposthenia of the right hemosome also appeared in the last days. He was originally from Egypt, specifically area of Asyut. The last time he visited his country was 6 months before, where he reported to have eaten unpasteurized dairy products and assisted to sheep and goat giving birth. On examination, he was alert, slow, and feverish. He reported headache. He had meningeal signs irritation, global aphasia, and decreased power in right lower limb. Laboratory exams showed white blood cells (WBCs) 6650/mmc (50% mononuclear) and C-reactive protein 60 mg/L.

Brain CT scan and RMN were normal. CSF examination showed high white blood cells with lymphocytes pleocytosis (WBC 519/mmc, lymphocytes 89%), high protein level (277 mg/dl), and decreased glucose (25 mg/dl, capillary glucose 93 mg/dl). Empiric therapy with ceftriaxone, ampicillin and acyclovir was introduced.

A few days later blood culture and CSF culture resulted positive for *Brucella melitensis* along with positive serology (IgA, IgG, and IgM). Meanwhile molecular biology for common viruses and bacteria was negative, as well as microscopic examination and culture for *Mycobacterium tuberculosis*.

Antimicrobial therapy was modified to ceftriaxone 2 gr q12h, rifampin 900 mg q24h, and doxycycline 100 mg q12h. Ceftriaxone was continued for two months, while it was decided to continue rifampin and doxycycline until the normalization of cellular and biochemical CSF parameters.

The patient was discharged in a discrete clinical condition two months later and was followed-up by our outpatient service, where he underwent several lumbar punctures to assess when to stop therapy (Table S2).

The patient’s clinical condition gradually improved, he gained weight and reported a normalization of mood tone.

He stopped medications in May 2023 on his own. Two months later he came back to the outpatient service: he was in good clinical conditions, no sequelae reported, and the lumbar puncture showed the normalization of CSF parameters. Therefore, the end of the treatment was confirmed.

### Systematic review and pooled analysis

#### Study characteristics, geographic origin, and quality assessment

**Fig. 1:**
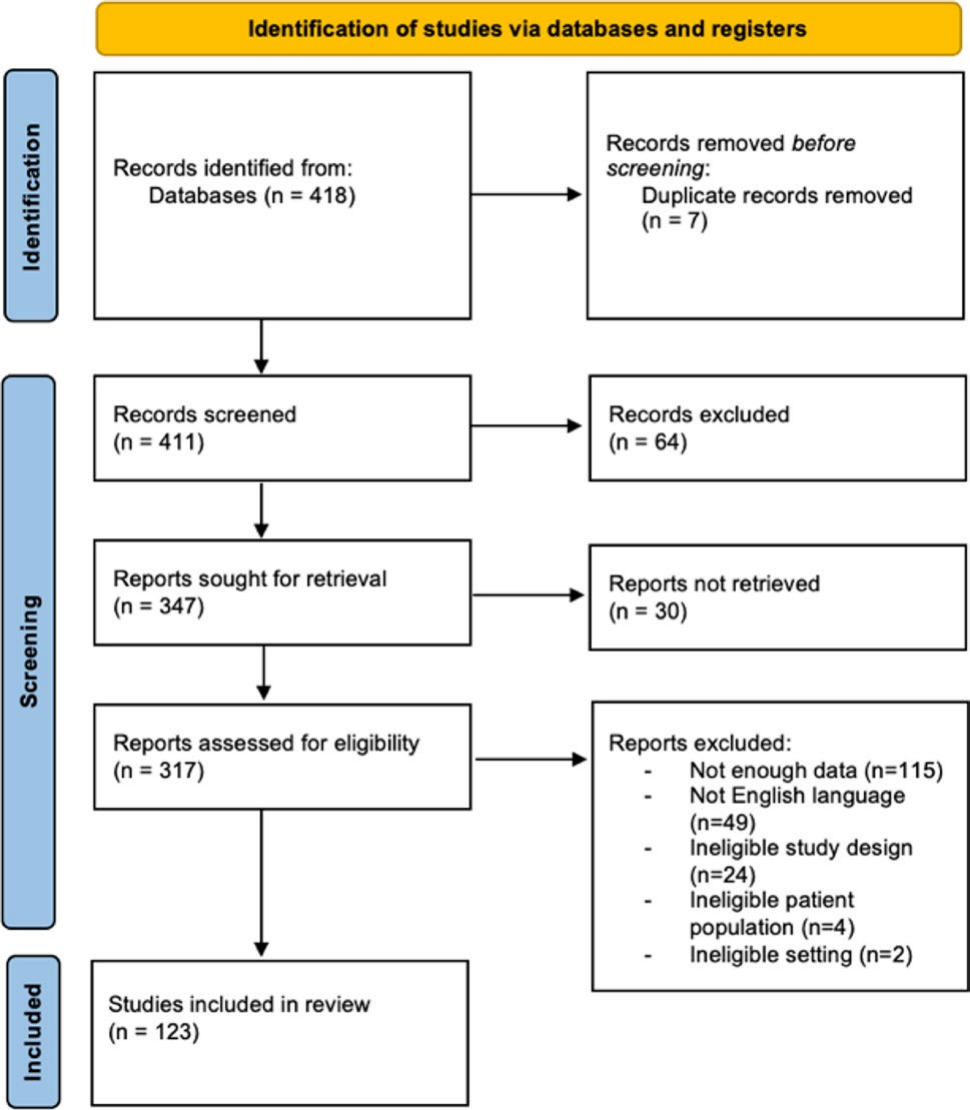
– PRISMA 2020 Flow Diagram for systematic reviews including database and registries

A total of 123 studies comprising 221 patients were included in this review (Fig. 1). One hundred were case report with just one patient, meanwhile 23 studies presented the experience of multiple patients with neurobrucellosis (for references see Supplementary materials). No randomized trial was included.

Predominantly, the studies were from countries endemic to brucellosis, notably 146/221 cases (66%) were from the Middle East. Among these, Turkey contributed the majority (84/146, 58%), followed by Saudi Arabia (34/146, 23%). Within the Mediterranean basin, cases were documented in Greece, Italy, Spain, and Portugal. Some of the included clinical cases were imported; notably, 5 out of 6 cases recorded in the USA pertained to migrants or travelers with a history of dairy product consumption in Central-South American or Middle Eastern countries. Similar cases of importation were also noted in Europe, particularly in Germany (Figure S1).

The methodological quality of the included studies is summarized in Supplementary data (Figure S2). Eighty-seven out of 123 studies were judged of good methodological quality, while 36/123 low methodological quality. Among these, the most critical domain was selection bias, due to publication bias and the type of design of the included studies.

#### Pooled analysis

##### Descriptive analysis

A total of 221 individual cases were included in the analysis. The overall median duration of treatment was 4 months (IQR 3 – 6), and similar values were obtained if the two populations (pediatric and adult) were considered separately. 152 patients (68.8%) recovered without sequelae, 5 patients had a relapse (2.3%) of the disease, and 4 patients (1.8%) died. In 59 cases (27.1%), there were some sequelae, including 25 patients with permanent hearing loss, 9 vision problems, 25 cases of persistent motor impairment, and 4 cases of cognitive dysfunction. The overall features of the included population are depicted in Table 1.

**Table 1:** Descriptive analysis: patient characteristics, signs and symptoms, clinical presentation, microbiological and radiological features, treatment, follow-up, and outcomes of the patients included in the final analysis

	Overall (N = 221)
Demographics Characteristics	
Provenance	
Middle East	146/221 (66)
Asia	34/221 (15)
Europe	29/221 (13)
Latin America	6/221 (3)
Africa	2/221 (1)
North America	1/221 (1)
Sex at birth: Female	76/221 (34)
Age (years)	31.0 (20.0, 47.8)
Pediatric cases	43/221 (19)
Previous Brucellosis	23/221 (10)
Symptoms	
Fever	144/221 (65)
Headache	132/221 (60)
Nausea or vomiting	69/221 (31)
Altered sensorium	56/221 (25)
Walking difficulty	48/221 (21)
Muscular weakness	43/221 (19)
Malaise or fatigue	42/221 (19)
Auditory impairment	34/221 (15)
Visual impairment	33/221 (15)
Weight loss or anorexia	32/221 (14)
Back pain	29/221 (13)
Psychiatric symptoms	28/221 (12)
Joint pain	24/221 (11)
Sweating	22/221 (10)
Dizziness	16/221 (7)
Myalgia	16/221 (7)
Sensitive alteration	13/221 (6)
Urinary incontinence	13/221 (6)
Urinary retention	10/221 (4)
Chills	7/221 (3)
Abdominal pain	5/221 (2)
Fecal incontinence	4/221 (2)
Other symptoms (miscellaneous)	22/221 (10)
Signs	
Motor deficit	90/221 (41)
Meningeal irritation signs	76/221 (34)
Decreased muscle strength	49/221 (22)
Confusion	41/221 (19)
Decreased deep tendon reflexes (DTR)	35/221 (16)
Senso-neural hearing loss	34/221 (15)
Hypoesthesia and Paresthesia	33/221 (15)
Increased DTR	29/221 (13)
Papilledema	29/221 (13)
Convulsions	24/221 (11)
Hemiparesis	20/221 (9)
Diplopia	20/221 (9)
Ataxia	20/221 (9)
Positive Babinski sign	20/221 (9)
Paraparesis	20/221 (9)
Dysarthria	15/221 (7)
Hepatosplenomegaly	14/221 (6)
Aphasia	9/221 (4)
Tremor	8/221 (4)
Positive Romberg test	7/221 (3)
Decreased visual acuity	7/221 (3)
Paraplegia	6/221 (3)
Nystagmus	4/221 (2)
Ascites	1/221 (1)
Onset of hearing loss (months) ^n=23^	12 (3.5, 13.5)
Onset of visual alteration (days) ^n=16^	10 (7.0, 67.5)
Onset of generic symptoms (months) ^n=132^	1.4 (0.5, 4.0)
Onset of psychiatric symptoms (months) ^n=9^	1.5 (1.0, 4.0)
Onset of neurological symptoms (days) ^n=140^	30 (7, 120)
Microbiological diagnosis	
Positive blood culture for *Brucella* spp.	50/199 (25)
Positive bone marrow culture for *Brucella* spp.	5/199 (2)
Positive blood serology assay for *Brucella* spp.	172/199 (86)
Positive blood PCR for *Brucella* spp.	1/199 (1)
Positive CSF culture for *Brucella* spp.	49/198 (25)
Positive abscess drainage culture for *Brucella* spp.	7/198 (3)
Positive CSF serology assay for *Brucella* spp.	136/198 (69)
Positive CSF PCR for *Brucella* spp.	12/198 (6)
CSF laboratory parameters	
Abnormal CSF parameters	206/209 (99)
Glucose alteration in CSF	149/209 (72)
Protein alteration in CSF	176/209 (85)
WBC alteration in CSF	178/209 (86)
Lymphocyte count alteration in CSF	133/209 (65)
Imaging	
Abnormal imaging	103/185 (56)
Vascular involvement	24/103 (23)
Leptomeningeal enhancement	23/103 (22)
Edema	14/103 (14)
Deep white matter hyperintensities (WMH)	13/103 (13)
Hydrocephalus	13/103 (13)
Periventricular WMH	12/103 (12)
Spinal root enhancement	9/103 (9)
Abscess	9/103 (9)
Granuloma	7/103 (7)
Basal meningeal enhancement	5/103 (5)
Cranial nerve enhancement	5/103 (5)
Demyelinating plaque	3/103 (3)
Leukoencephalopathy	2/103 (2)
Arachnoiditis	1/103 (1)
Clinical presentation	
Meningitis	101/221 (46)
Cranial nerve involvement	47/221 (21)
Meningoencephalitis	26/221 (11)
Radiculopathy	23/221 (10)
Stroke	21/221 (10)
Increased intracranial pressure	17/221 (8)
Peripheral nervous system involvement*	15/221 (7)
Myelitis	10/221 (5)
CNS abscess	8/221 (4)
Hydrocephalus	8/221 (4)
CNS granulomas	7/221 (3)
Ventriculoperitoneal shunt infection	6/221 (3)
Vasculitis	6/221 (3)
SIADH	4/221 (2)
Arachnoiditis	3/221 (1)
Upper motor neuron involvement	2/221 (1)
Sinus thrombosis	2/221 (1)
Diabetes insipidus	1/221 (1)
Subdural empyema	1/221 (1)
Mycotic aneurysm	1/221 (1)
Treatment	
Duration (months) ^n=214^	4 (3–6)
Use of ceftriaxone	105/211 (50%)
Use of corticosteroids	37/221 (17)
Control lumbar puncture at end of therapy performed	53/181 (29)
Abnormal CSF parameters	15/53 (28)
Outcomes	
Recovery without sequelae	152/221 (69)
Recovery with sequelae	60/221 (27)
Relapse	5/221 (2)
Death	4/221 (2)
Sequelae	
Sensorineural hearing loss	25/60 (42)
Motor deficit	25/60 (42)
Abnormal vision	9/60 (15)
Cognitive dysfunction	4/60 (7)
Other	7/60 (12)

Continuous variables are reported as median (quartile 1 to quartile 3) and categorical variables are presented as number (percentage)

^*^Peripheral nervous system involvement comprised: isolated peripheralnerve involvement (12/221, 5%), peripheral neuropathy (4/221, 2%), Guillain-Barrè syndrome (3/221, 1%)

##### Univariate and multivariable analyses

In the univariate analysis, hearing loss, motor impairment, and duration of symptoms > 3 months at presentation were significantly associated with a higher risk of relapse or sequalae. On the contrary, length of antimicrobial treatment was not associated, whether expressed as a continuous (5.4 months *versus* 4.9, p = 5.04) or as categorical (< *versus* ≥ 6 months) variable (34/65 *versus* 60/152, p = 0.11) (Table 2).

**Table 2:** Univariate analysis assessing the characteristics of the patients included in the final analysis stratified by recovery status

Variable	No Recovery (N = 65)	Recovery (N = 152)	P value
Mean age (SD)	37.6 (18.2)	32.0 (18.0)	0.39
Mean treatment duration (SD)	5.4 (3.7)	4.9 (3.5)	5.04
6 months of treatment (%)	34 (52)	60 (40)	0.11
Use of ceftriaxone (%)	29 (45)	76 (51)	0.57
Use of corticosteroids (%)	18 (28)	19 (12)	0.11
Motor deficit (%)	42 (65)	46 (30)	< 0.001
Hearing loss (%)	30 (46)	4 (3)	< 0.001
Onset of symptoms < 3 months (%)	33 (62)	28 (30)	< 0.001
Pediatric cases (%)	9 (14)	35 (23)	2.55

N is the number of patients with available information for each variable. SD = standard deviation

The main multivariate analysis found that corticosteroids use (OR 0.39, 95% IC 0.16 – 0.96, p = 0.038), motor impairment (OR 0.29, 95% IC 0.14 – 0.62, p = 0.002) and hearing loss (OR 0.037, 95% IC 0.01 – 0.11, p < 0.001) were significantly associated with a higher risk of relapse or sequalae. No difference was observed for the age of patients, the duration of treatment (continuous variable), and the use of ceftriaxone between the two groups (Table 3). These results were confirmed when the duration of treatment was considered as a categorical variable (Table S3). Corticosteroids use and hearing loss were found to be associated with the outcome in the secondary multivariable analysis (Table S4), also including the duration of symptoms as categorical variable. Moreover, the sensitivity analysis, excluding pediatric patients, confirmed that motor impairment and hearing loss were statistically associated with the outcome (Table 4).

**Table 3:** Multivariable Analysis assessing factors associated with a poor recovery status

Variable	Recovery aOR (95% CI)	P value
Age	0.99 (0.97–1.01)	0.36
Treatment duration	1.02 (0.92–1.14)	0.66
Use of ceftriaxone	0.91 (0.42–1.94)	0.81
Use of corticosteroids	0.38 (0.16–0.96)	0.038
Motor deficit	0.29 (0.14–0.62)	0.002
Hearing loss	0.04 (0.01–0.11)	< 0.001

aOR = adjusted odds ratio; CI = confidence interval

**Table 4:** Sensitivity Analysis assessing factors associated with a poor recovery status by excluding pediatric patients (n = 43/221)

Variable	Recovery aOR (95% CI)	P value
Age	1 (0.97–1.02)	0.76
Treatment duration	1.06 (0.94–1.2)	0.36
Use of ceftriaxone	0.93 (0.39–2.14)	0.86
Use of corticosteroids	0.43 (0.15–1.31)	0.13
Motor deficit	0.23 (0.1–0.54)	0.001
Hearing loss	0.04 (0.01–0.11)	< 0.001

aOR = adjusted odds ratio; CI = confidence interval

The CoE for the primary outcome was deemed to be at very low certainty due to the inclusion of case reports and case series alone.

## Discussion

Our literature review including 221 cases of neurobrucellosis highlights that the median treatment duration was 4 months (IQR 3 – 6). Treatment duration ≥ 6 months was not statistically correlated with a different risk of sequelae or relapse, which occurred in 29% of patients. Instead, factors associated with an unfavorable outcome in our main multivariate analysis were the presence of motor impairment, hearing loss, and use of corticosteroids.

Literature lacks conclusive evidence on treatment duration, with conflicting recommendations ranging from approximately 6 to 8 weeks to a median of 6 months. Despite these suggestions, most authors recommend continuing treatment until the normalization of CSF chemical-physical parameters, making treatment duration challenging to standardize [2, 3]. However, despite this recommendation, monitoring of CSF parameters is not frequent. Among the case reports included in this review, lumbar puncture control was reported in only 53 out of 187 cases (28%), and treatment was discontinued at the normalization of CSF chemical-physical parameters in only 38 cases (20%). Regarding therapeutic regimens, recent literature review and an older retrospective study in Turkey highlighted that antibiotic therapy involving ceftriaxone was associated with significantly shorter treatment in the adult population [3, 7]. This was not evident in the pediatric population [3]. Contrarily, in our study, no such difference in duration emerged, considering both the overall population and stratifying by pediatric and adult age groups. In fact, the average treatment duration was statistically longer for regimens with ceftriaxone compared to the regimens without (mean 5.7 ± 3.7 *versus* 4.5 ± 3.5 months; p = 0.014). This could be attributed to selection bias, as the choice to use ceftriaxone may be linked to more severe clinical presentations.

The different result with the aforementioned literature review could be credited to the fact that we divided the patients between therapy with *versus* without ceftriaxone, while Tajerian and collegues [3] between “ceftriaxone with the supplementation of standard oral regimens” *versus* “standard oral antibiotic regimens”. Furthermore, no significant differences were observed between the two groups regarding ceftriaxone use, contrary to previous literature evidence associating ceftriaxone with significantly fewer relapses or therapeutic failures [7].

In the main multivariate analysis, motor damage and hearing loss were confirmed to be associated with a higher risk of sequelae or relapse, a finding consistent even when considering only adult patients.

Hearing loss is a quite common complication of neurobrucellosis, as well as of other forms of bacterial meningitis. The deficit may be due to direct damage to the nerve caused by bacterial invasion, to a demyelination process, or it could be secondary to a condition of tissue hypoxia [8]. The way in which hearing loss is established easily explains why it is mostly irreversible or only partially associated with mild to moderate auditory recovery in most cases.

There is no significant literature evidence on the association of motor deficits and clinical outcome. However, the correlation between this clinical presentation and the risk of sequelae could be attributed to damage to the motor cortex, as indicated by a higher percentage of signs and symptoms correlated with involvement of the first motor neuron. These symptoms are generally described as mild in the early stages of the disease and with a progressive course, which could further support the risk of sequelae associated with this manifestation due to delayed access to treatment.

The use of corticosteroid therapy was also statistically associated with a higher risk of sequelae or relapse in the main multivariate analysis. It is known that the use of steroids in Pneumococcal meningitis reduces the risk of neurological sequelae, including hearing loss. Some cohort studies have allowed extending this concept to all bacterial etiologies of meningitis, excluding *Listeria* spp. meningitis, where the use of corticosteroids was associated with a higher mortality rate [9–11]. This difference could be attributed to some unique characteristics of *Listeria* spp. meningitis compared to other bacterial meningitides, including a more subacute onset and more frequent meningoencephalitic involvement and cranial nerve impairment, features shared with neurobrucellosis.

Importantly, the protective effect of steroids emerged from studies conducted in high-income countries, while no difference was observed in their use in countries with a low level of medical care [11, 12].

The negative effect of steroids on the outcome may be influenced by their use in more critical cases in our study. Despite this, the current evidence weighs negatively against their use in neurobrucellosis and underscores the need for further studies to clarify their actual effect in this disease.

Our study has some limitations. We included only case reports and case series, as we did not find comparative studies that would allow for stronger inferential analyses. Despite this limitation, it is essential to highlight that, for rare conditions as neurobrucellosis, resorting to a systematic review can help gather available evidence.

Secondly, the inclusion of case reports and series is subject to publication bias.

Third, the different lengths of follow-up and the varied ways of reporting it expose the study to informative censoring and related biases.

Finally, the inclusion criterion based on treatment duration does not allow for a comprehensive consideration of other emerging evidence. However, the diagnostic criteria that we used to select cases with this condition were highly stringent, therefore reducing reporting, causality, and ascertainment biases.

## Conclusions

Neurobrucellosis is a low-frequency disease and diagnosis is often delayed due to insidious clinical presentation and low clinical suspicion, along with the poor yield of cultures from cerebrospinal fluid and blood.

This further underscores the importance of considering this diagnosis in patients from or traveling to endemic areas for brucellosis with exposure to risk factors, to ensure timely and appropriate treatment, thus potentially reducing the risk of disease sequelae.

In our review, treatment duration longer than 6 months was not associated with a lower risk of relapse or sequalae, which seemed to be more linked to the type of symptoms at presentation (motor impairment and hearing loss) and to the use of corticosteroids. However, given the type of studies included, we are uncertain on giving recommendations based on these results.

This highlights the need for additional studies enrolling consecutive patients to better understand this condition and provide more precise indications regarding the treatment and management of patients affected by this disease.

## Supplementary Information

Below is the link to the electronic supplementary material.

Supplementary file1 (DOCX 175 KB)

## Data Availability

The datasets generated during and/or analysed during the current study are available from the first or corresponding author on reasonable request.
