# Supplementary material for: Neurobrucellosis Presenting with Motor Damage or Hearing Loss, and Use of Steroids are Associated with a Higher Risk of Sequelae or Relapse: A Systematic Review of Individual Participant Data
Source: Neurol Sci. 2024 Jun 11;45(11):5441–8. doi: 10.1007/s10072-024-07621-6 (PMC11470871; doi:10.1007/s10072-024-07621-6)

**Supplementary data legend**

1. Additional references of the studies included in the systematic review
2. **Table S1** – Search term
3. **Table S2** – Cerebrospinal fluid parameters during patient’s treatment
4. **Table S3** – Multivariable Analysis assessing assessing factors associated with a poor recovery status considering the duration of treatment as a categorical variable (>6 months) **Table S4** – Multivariable secondary analysis assessing factors associated with a poor recovery status considering the onset of symptoms as a categorical variable (> 3 months).
5. **Table S5** – PRISMA 2020 Checklist
6. **Figure S1** – Methodological assessment
7. **Figure S2** - Geographical distribution of the individual cases reviewed, according to their numerosity by country of diagnosis

**Additional references of the studies included in the systematic review**

1. Abuzinadah, A. R. *et al.* Brucellosis causing subacute motor polyradiculopathy and the pathological correlation of pseudomyopathic electromyography: A case report. *Clin. Neurophysiol. Pract.* **5**, 130–134 (2020).
2. Aghamollaii, V., Ahmadinejad, Z., Mohammadian, F. & Mirsepassi, Z. Catatonic state as a rare presentation of neurobrucellosis: A case report. *Iran. J. Psychiatry Behav. Sci.* **13**, 10–12 (2019).
3. Akçam, F. Z. *et al.* Neurobrucellosis: Two cases, two different presentation. *Turk Noroloji Derg.* **26**, 342–345 (2020).
4. Akcay, H., Kurtuncu, M., Celik, S. Y., Gunduz, T. & Eraksoy, M. Neurobrucellosis presenting with mania. *Acta Neurol. Belg.* **117**, 373–375 (2017).
5. Akdeniz, H., Irmak, H., Anlar, Ö. & Demiröz, A. P. Central nervous system brucellosis: Presentation, diagnosis and treatment. *J. Infect.* **36**, 297–301 (1998).
6. Akhondian, J., Ashrafzadeh, F., Beiraghi Toosi, M. & Hashemi, N. A Rare presentation of neurobrucellosis in a child with Recurrent transient ischemic attacks and pseudotumor cerebri (A case report and review of literature). *Iran J Child Neurol.* (2014).
7. Akin, A. *et al.* A case of neurobrucellosis. *J. Exp. Clin. Med.* **32**, 39–41 (2015).
8. Aktham, A. D. *et al.* A Cluster of Neurobrucellosis at King Hussein Medical Center, Jordan - A Comprehensive Analysis And Review. *Int. Res. J. Sci. Technol.* **2**, 349–358 (2020).
9. Al-Eissa, Y. A. Clinical and therapeutic features of childhood neurobrucellosis. *Scand. J. Infect. Dis.* **27**, 339–343 (1995).
10. Al-Otaibi, A., Almuneef, M. & Shaalan, M. Al. Brucella melitensis infection of ventriculo-peritoneal shunt: A form of neurobrucellosis manifested as gastrointestinal symptoms. *J. Infect. Public Health* **7**, 62–65 (2014).
11. Al-Qarhi, R. & Al-Dabbagh, M. Brucella shunt infection complicated by peritonitis: Case report and review of the literature. *Infect. Dis. Rep.* **13**, 367–376 (2021).
12. Ali Alanazi, A. A. *et al.* Acute brucellosis with a guillain-barre syndrome-like presentation: A case report and literature review. *Infect. Dis. Rep.* **13**, 1–10 (2021).
13. Alqwaifly, M., Al-Ajlan, F. S., Al-Hindi, H. & Semari, A. Al. Central nervous system brucellosis granuloma and white matter disease in immunocompromised patient. *Emerg. Infect. Dis.* **23**, 978–981 (2017).
14. Altas, M., Evirgen, O., Arica, V. & Tutanc, M. Pediatric neurobrucellosis associated with hydrocephalus. *J. Pediatr. Neurosci.* **5**, 144–146 (2010).
15. Amiri, R. S., Hanif, H., Ahmadi, A. & Amirjamshidi, A. Brucella-related multiple cerebral aneurysms: Report of a case and review of the literature. *Surg. Neurol. Int.* **5**, 1–5 (2014).
16. Andersen, H. & Mortensen, A. Unrecognised Neurobrucellosis Giving Rise to Brucella melitensis Peritonitis Via a Ventriculoperitoneal Shunt. *Eur. J. Clin. Microbiol. Infect. Dis.* **11**, 953–954 (1992).
17. Ay, S., Tur, B. S. & Kutlay, Ş. Cerebral infarct due to meningovascular neurobrucellosis: A case report. *Int. J. Infect. Dis.* **14**, 2009–2011 (2010).
18. Yazdi, N. A., Moosavi, N. S., Alesaeidi, S., Salahshour, F. & Ghaemi, O. Diffuse Neurobrucellosis of Cerebellum, Brainstem, Spinal Cord, and Cauda Equina: A case report and Literature review. *J. Radiol. Case Rep.* **16**, 1–9 (2022).
19. Babamahmoodi, F. & Babamahmoodi, A. Brucellosis, presenting with guillain-barré syndrome. *J. Glob. Infect. Dis.* **3**, 390–392 (2011).
20. Bains, R. *et al.* An Interesting Case of Neurobrucellosis Mimicking Neuropsychiatric Lupus. *Case Rep. Rheumatol.* **2018**, 1–5 (2018).
21. Banerjee, T. K., Pal, A. K. & Das, S. Neurobrucellosis presenting as acute meningoencephalitis. *Neurol. India* **47**, 160 (1999).
22. Bessisso, M. S., Elsaid, M. F., Elshazli, S. S. E. & Hatim, M. Case Report Neuro-brucellosis in children. 2000–2002 (2000).
23. Bingöl, A. & Togay-Işikay, C. Neurobrucellosis as an exceptional cause of transient ischemic attacks. *Eur. J. Neurol.* **13**, 544–548 (2006).
24. Bodur, H. *et al.* Neurobrucellosis in an endemic area of brucellosis. *Scand. J. Infect. Dis.* **35**, 94–97 (2003).
25. Bouza, E. *et al.* Brucellar meningitis. *Rev. Infect. Dis.* **9**, 810–822 (1987).
26. Bussone, G. *et al.* Neurobrucellosis Mimicking Multiple Sclerosis: A Case Report. *Eur Neurol* (1989).
27. Cagatay, A., Karadeniz, A., Ozsut, H., Eraksoy, H. & Calangu, S. Hearing loss in patient with neurobrucellosis [2]. *South. Med. J.* **99**, 1305–1306 (2006).
28. Çiftçi, E., Erden, I. & Akyar, S. Brucellosis of the pituitary region: MRI. *Neuroradiology* **40**, 383–384 (1998).
29. Covrig, R. C., Petridou, J. & Knappe, U. J. Atypical Presentation of Neurobrucellosis as Infection of Ventriculoperitoneal Shunt. *J. Neurol. Surgery, Part A Cent. Eur. Neurosurg.* **82**, 186–188 (2021).
30. Daglioglu, E. *et al.* Lumbar epidural abscess caused by brucella species: Report of two cases. *Neurocirugia* **20**, 159–162 (2009).
31. Dar, W., Latief, M., Dar, I. & Sofi, N. Meningitis, polyradiculopathy, and optic nerve involvement in neurobrucellosis: A rare clinical presentation. *Neurol. India* **65**, 1142–1144 (2017).
32. Dar, W. *et al.* Neurobrucellosis - Overlooked but Treatable Cause of Hearing Loss. *Neurol. India* **69**, 1893–1894 (2021).
33. Despotopoulos, S., Akinosoglou, K., Tzimas, T., Akritidis, N. & Gogos, C. Diagnosis of neurobrucellosis in resource-limited real-world settings: A case-series of 8 patients. *J. Neurol. Sci.* **379**, 293–295 (2017).
34. Diaz Espejo, C. E., Chaves, F. V. & Ramis, B. S. Chronic intracranial hypertension secondary to neurobrucellosis. *J. Neurol.* **234**, 59–61 (1987).
35. Díaz-Vintimilla, J. J., Rosales Hernández, L. A., Zapata-Arenas, R., Sánchez-Montes, S. & Becker, I. Subacute transverse myelitis as a clinical presentation of neurobrucellosis. *J. Infect. Dev. Ctries.* **15**, 1359–1363 (2021).
36. Estevão Lopes, M. H., Barroso Winckler, L. M. F., Matos, L. M. A. F. D., Barroso, A. A. & Carmona da Mota, H. Neurobrucellosis in children. *Eur J Pediatr* (1995). doi:10.1016/S2221-1691(15)30160-X
37. Farhan, N., Khan, E. A., Ahmad, A. & Ahmed, K. S. Neurobrucellosis: A report of two cases. (2017).
38. Ghosh, D., Gupta, P. & Prabhakar, S. Systemic Brucellosis with chronic meningitis: A case report. *Neurol. India* **47**, 58–60 (1999).
39. Gokul, B. N., Paul, A. & Hussein, I. Neurobrucellosis. **966**, 182–185
40. Habeeb, Y. K. R., Ai-Najdi, A. K. N., Sadek, S. A. H. & Al-Onaizi, E. Paediatric neurobrucellosis: Case report and literature review. *J. Infect.* **37**, 59–62 (1998).
41. Hadda, V., Khilnani, G. C. & Kedia, S. Brucellosis presenting as pyrexia of unknown origin in an international traveller: A case report. *Cases J.* **2**, 1–4 (2009).
42. Havalı, C. & Çağan, E. A rare pediatric case of neurobrucellosis with bilateral optic neuritis. *Turk. J. Pediatr.* **62**, 1094–1098 (2020).
43. Hesseling, A. C., Marais, B. J. & Cotton, M. F. A child with neurobrucellosis. *Ann. Trop. Paediatr.* **23**, 145–148 (2003).
44. Inan, A. S. *et al.* Neurobrucellosis with transient ischemic attack, vasculopathic changes, intracerebral granulomas and basal ganglia infarction: a case report. *J. Med. Case Rep.* **4**, 1–5 (2010).
45. Jabbour, R. A. & Tabbarah, Z. Neurobrucellosis presenting with hearing loss, gait disturbances and diffuse white matter disease on brain magnetic resonance imaging (MRI). *J. Med. Liban.* **59**, 105–108 (2011).
46. Jochum, T., Kliesch, U., Both, R., Leonhardi, J. & Bär, K. J. Neurobrucellosis with thalamic infarction: A case report. *Neurol. Sci.* **29**, 481–483 (2008).
47. Kim, E. J. *et al.* Relapsed brucellosis presenting as neurobrucellosis with cerebral vasculitis in a patient previously diagnosed with brucellar spondylitis: A case report. *Infect. Chemother.* **47**, 268–271 (2015).
48. Kale, G. *et al.* Neurobrucellosis: A short case series. *Ann. Indian Acad. Neurol.* **20**, S28 (2017).
49. Kanjo, M. A., Ahmed, H. M. & Alnahari, E. A. Unusual presentation of neurobrucellosis in Jeddah, Kingdom of Saudi Arabia. *Neurosciences* **26**, 385–388 (2021).
50. Karaca, S., Demiroglu, Y. Z., Karataş, M. & Tan, M. Acquired progressive spastic paraparesis due to neurobrucellosis: A case report. *Acta Neurol. Belg.* **107**, 118–121 (2007).
51. Kesav, P., Modi, M., Singla, V., Khurana, D. & Prabhakar, S. Kaleidoscopic presentation of neurobrucellosis. *J. Neurol. Sci.* **331**, 165–167 (2013).
52. Kizilkilic, O. *et al.* Successful medical treatment of intracranial abscess caused by Brucella spp. *J. Infect.* **51**, 77–80 (2005).
53. Kochlar, D. K. *et al.* Meningoencephalitis in brucellosis. *Neurol. India* **48**, 170–173 (2000).
54. Korkmaz, P. *et al.* A case of neurobrucellosis detected during brucella treatment. *Ann. Clin. Anal. Med.* **12**, 1188–1190 (2021).
55. Köse, Ş. *et al.* Clinical manifestations, complications, and treatment of brucellosis: Evaluation of 72 cases. *Turkish J. Med. Sci.* **44**, 220–223 (2014).
56. Krishnan, C., Kaplin, A. I., Graber, J. S., Darman, J. S. & Kerr, D. A. Recurrent transverse myelitis following neurobrucellosis: Immunologic features and beneficial response to immunosuppression. *J. Neurovirol.* **11**, 225–231 (2005).
57. Lemnouer, A. *et al.* Brucellosis: A cause of meningitis not to neglect. *IDCases* **10**, 97–99 (2017).
58. Levy, J., Shneck, M., Marcus, M. & Lifshitz, T. Brucella meningitis and papilledema in a child. *Eur. J. Ophthalmol.* **15**, 818–820 (2005).
59. Li, Q. *et al.* A case of brucellosis-induced Guillain–Barre syndrome. *BMC Infect. Dis.* **22**, 1–5 (2022).
60. Mahdavi, F. S., Abbasi Khoshsirat, N. & Madanipour, A. Lumbosacral polyradiculitis associated with brucellosis. *IDCases* **23**, e01028 (2021).
61. Marques, R. *et al.* Unilateral optic neuritis as a presentation of neurobrucellosis. *Pediatr. Rep.* **3**, 11–12 (2011).
62. Martínez-Chamorro, E., Muñoz, A., Esparza, J., Muñoz, M. J. & Giangaspro, E. Focal cerebral involvement by neurobrucellosis: Pathological and MRI findings. *Eur. J. Radiol.* **43**, 28–30 (2002).
63. McLean, D. R., Russell, N. & Yousuf Khan, M. Neurobrucellosis: Clinical and therapeutic features. *Clin. Infect. Dis.* **15**, 582–590 (1992).
64. Mehta, S. & Mehta, S. Neurobrucellosis presented with hemorrhagic stroke: A rare case report. *Int. J. Infect. Dis.* **101**, 168 (2020).
65. Miguel, P. S. *et al.* Neurobrucellosis mimicking cerebral tumor: case report and literature review. *Clin. Neurol. Neurosurg.* **108**, 404–406 (2006).
66. Militão, A., Semedo, C. & Matos, R. Neurobrucellosis: A case of reversible rapidly progressive dementia. *Eur. J. Neurol.* **24**, 422–422 (2017).
67. Mohammadzadeh, V., Akbarieh, S., Ghoreishi, S. A. & Jozpanahi, M. Neurobrucellosis with intramedullary spinal cord involvement: A case report. *Curr. J. Neurol.* **17**, 152–153 (2019).
68. Nas, K. *et al.* Cervical intramedullary granuloma of Brucella: A case report and review of the literature. *Eur. Spine J.* **16**, 255–259 (2007).
69. Novati, R. *et al.* Neurobrucellosis with spinal cord abscess of the dorsal tract: A case report [3]. *Int. J. Infect. Dis.* **6**, 149–150 (2002).
70. Abu-Omar, Y. *et al.* European Association for Cardio-Thoracic Surgery expert consensus statement on the prevention and management of mediastinitis. *Eur. J. Cardio-thoracic Surg.* **51**, 10–29 (2017).
71. Özisik, H. I., Ersoy, Y., Refik Tevfik, M., Kizkin, S. & Özcan, C. Isolated intracranial hypertension: A rare presentation of neurobrucellosis. *Microbes Infect.* **6**, 861–863 (2004).
72. Papadopoulos, V. E. *et al.* Seronegative neurobrucellosis—do we need new neurobrucellosis criteria? *Int. J. Infect. Dis.* **111**, 124–126 (2021).
73. Patra, S., Kalwaje Eshwara, V., Pai, A. R., Varma, M. & Mukhopadhyay, C. Evaluation of clinical, diagnostic features and therapeutic outcome of neurobrucellosis: a case series and review of literature. *Int. J. Neurosci.* **132**, 1080–1090 (2022).
74. Paydarnia, P., Moradi, S., Habibi, A., Abbasian, L. & Ghabaee, M. A case report of neurobrucellosis mimicking Guillain–Barré syndrome. *Neurol. Psychiatry Brain Res.* **31**, 27–28 (2019).
75. Rajan, R., Khurana, D. & Kesav, P. Teaching NeuroImages: Deep gray matter involvement in neurobrucellosis. *Neurology* **80**, 28–30 (2013).
76. Ahmed, R. & Patil, B. S. Neurobrucellosis: A rare cause for spastic paraparesis. *Brazilian J. Infect. Dis.* **13**, 245 (2009).
77. Powers, H. R., Nelson, J. R., Alvarez, S. & Mendez, J. C. Neurobrucellosis associated with feral swine hunting in the southern United States. *BMJ Case Rep.* **13**, 13–16 (2020).
78. Rossi, M. *et al.* Neurobrucellosis: Diagnostic and clinical management of an atypical case. *New Microbiol.* **41**, 165–167 (2018).
79. Miyares, F. R. *et al.* Irreversible papillitis and ophthalmoparesis as a presenting manifestation of neurobrucellosis. *Clin. Neurol. Neurosurg.* **109**, 439–441 (2007).
80. Samdani, P. G. & Patil, S. Neurobrucellosis. *Indian Pediatr.* (2003).
81. Sarmiento Clemente, A., Amerson-Brown, M. H. & Foster, C. E. An Adolescent With Neurobrucellosis Caused by Brucella abortus Cattle Vaccine Strain RB51. *Pediatr. Infect. Dis. J.* **40**, E353–E355 (2021).
82. Sathyanarayanan, V., Ragini, B., Razak, A. & Prabhu, M. M. prana. Brucella meningoencephalitis with hydrocephalus masquerading as tuberculosis. *Asian Pac. J. Trop. Med.* **3**, 835–837 (2010).
83. Shah, I., Kawoos, Y., Sanai, B., Rabyang, S. & Banday, D. Neurobrucellosis presenting as acute psychosis. *J. Neurosci. Rural Pract.* **9**, 644–646 (2018).
84. Sheybani, F., Sarvghad, M. R., Bojdi, A. & Naderi, H. R. Brucellar psychosis. *Arch. Iran. Med.* **15**, 69–79 (2012).
85. Shirin, S., Abdollah, K., Alireza, F., Hassan, T. & Shahnaz, A. Rare presentations of neurobrucellosis. *Pakistan J. Med. Sci.* **24**, 464–467 (2008).
86. Shoshan, Y., Maayan, S., Gomori, M. J. & Israel, Z. Chronic subdural empyema: A new presentation of neurobrucellosis. *Clin. Infect. Dis.* **23**, 400–401 (1996).
87. Showkat, H. I. *et al.* Neurobrucellosis with bilateral sensorineural hearing loss and ataxia: A case report. *Schweizer Arch. fur Neurol. und Psychiatr.* **163**, 226–227 (2012).
88. Sohn, A. H. *et al.* Human neurobrucellosis with intracerebral granuloma caused by a marine mammal Brucella spp. *Emerg. Infect. Dis.* **9**, 485–488 (2003).
89. Spyrou, A., Natsis, K. S., Papamichalis, E. & Mourtzinos, H. Intraventricular haemorrhage and seizures in a patient with dementia: A case of chronic neurobrucellosis. *Age Ageing* **48**, 601–602 (2019).
90. Sturniolo, G. *et al.* Neurobrucellosis associated with syndrome of inappropriate antidiuretic hormone with resultant diabetes insipidus and hypothyroidism. *J. Clin. Microbiol.* **48**, 3806–3809 (2010).
91. Tekin-Koruk, S., Duygu, F., Gursoy, B., Karaagac, L. & Bayraktar, M. A rare case of seronegative neurobrucellosis. *Ann. Saudi Med.* **30**, 412–414 (2010).
92. Thomas, R., Kameswaran, M., Murugan, V. & Okafor, B. C. Sensorineural hearing loss in neurobrucellosis. *J. Laryngol. Otol.* (1993).
93. Trifiletti, R. R., Restivo, D. A., Pavone, P., Giuffrida, S. & Parano, E. Diabetes insipidus in neurobrucellosis. *Clin. Neurol. Neurosurg.* **102**, 163–165 (2000).
94. Tsyba, E., Gallego-Colon, E., Daum, A. Z., Fishman, E. & Chaim, Y. Pacemaker lead endocarditis: A rare cause of relapsing brucellosis. *IDCases* **13**, e00431 (2018).
95. Türel, Ö. *et al.* Acute meningoencephalitis due to brucella: Case report and review of neurobrucellosis in children. *Turk. J. Pediatr.* **52**, 426–429 (2010).
96. Ulas, U. H. *et al.* Paraplegia associated with brucellosis involving the anterior lumbrosacral nerve roots. *J. Peripher. Nerv. Syst.* **8**, 8–12 (2003).
97. Vafaeimanesh, J., Shahamzeh, A. & Bagherzadeh, M. Neurobrucellosis in systemic lupus erythematosus. *Casp. J. Intern. Med.* **8**, 119–122 (2017).
98. Vajramani, G. V., Nagmoti, M. B. & Patil, C. S. Neurobrucellosis presenting as an intra-medullary spinal cord abscess. *Ann. Clin. Microbiol. Antimicrob.* **4**, 1–5 (2005).
99. Villalobos-Vindas, J. M. *et al.* Brucellosis caused by the wood rat pathogen Brucella neotomae: Two case reports. *J. Med. Case Rep.* **11**, 1–4 (2017).
100. Vinod, P., Singh, M., Garg, R. & Agarwal, A. Extensive meningoencephalitis, retrobulbar neuritis, and pulmonary involvement in a patient of neurobrucellosis. *Neurol. India* **55**, 157–159 (2007).
101. Wen, H., Jin, D., Cai, L., Wu, T. & Liu, H. Neurobrucellosis with ischemic stroke and spinal cord involvement: a case report. *BMC Neurol.* **21**, 1–5 (2021).
102. Yamout, B. I., Massouh, J., Hushaymi, I., Zeineddine, M. & Saab, G. Neurobrucellosis presenting as longitudinally extensive transverse myelitis: A case report and review of the literature. *Mult. Scler. Relat. Disord.* **40**, 101947 (2020).
103. Anlar, Y. F., Yalcin, S. & Secmeer, G. Persistent Hypoglycorrhachia in Neurobrucellosis. (1994).
104. Yasin, M. & Moghtader Mojdehi, A. H. Acute neurobrucellosis: Syndrome of inappropriate antidiuretic hormone secretion and parkinsonism presentation. *Arch. Clin. Infect. Dis.* **9**, 3–5 (2014).
105. Yetkin, M. A., Bulut, C., Erdinc, F. S., Oral, B. & Tulek, N. Evaluation of the clinical presentations in neurobrucellosis. *Int. J. Infect. Dis.* **10**, 446–452 (2006).
106. Yilmaz, M. *et al.* Epileptic seizure: An atypical presentation in an adolescent boy with neurobrucellosis. *Scand. J. Infect. Dis.* **34**, 623–625 (2002).
107. Yilmaz, S., Serdaroglu, G., Gokben, S. & Tekgul, H. A case of neurobrucellosis presenting with isolated intracranial hypertension. *J. Child Neurol.* **26**, 1316–1318 (2011).
108. Zhang, J. *et al.* Treatment of a subdural empyema complicated by intracerebral abscess due to Brucella infection. *Brazilian J. Med. Biol. Res.* **50**, 1–4 (2017).
109. Işıkay, S., Yılmaz, K. & Ölmez, A. Neurobrucellosis developing unilateral oculomotor nerve paralysis. *Am. J. Emerg. Med.* **30**, 2085.e5-2085.e7 (2012).
110. Köse, H., Temoçin, F. & Temoçin, S. Neurobrucellosis Accompanied by Sternoclavicular Arthritis: A Case Report. *Haseki Tip Bul.* **55**, 322–324 (2017).
111. Turel, O. *et al.* A Rare Presentation of Neurobrucellosis in a 6-Year-Old Pediatric Patient with Sagittal Sinus Thrombosis. (2020).
112. Ertem, G., Kutlu, G., Hatipoǧlu, Ç. A., Bulut, C. & Demiröz, A. P. A rare presentation of brucellosis: polyradiculopathy and peripheral neuritis. *Turkish J. Med. Sci.* **42**, 359–364 (2012).
113. Lima, J. I. da S., Canelas, C. F. G., Veiga, A. S. de S. B. T. & Carvalho, D. M. M. Neurobrucellosis and venous sinus thrombosis: An uncommon association. *Rev. Soc. Bras. Med. Trop.* **49**, 383–385 (2016).
114. Fatani, D. F., Alsanoosi, W. A., Badawi, M. A. & Thabit, A. K. Ceftriaxone use in brucellosis: A case series. *IDCases* **18**, e00633 (2019).
115. Gul, H. C. *et al.* Management of neurobrocellosis: An assessment of 11 cases. *Intern. Med.* **47**, 995–1001 (2008).
116. Gündüz, T. *et al.* Characteristics of isolated spinal cord involvement in neurobrucellosis with no corresponding MRI activity: A case report and review of the literature. *J. Neurol. Sci.* **372**, 305–306 (2017).
117. Mahajan, S. K. *et al.* Neurobrucellosis: An often forgotten cause of chronic meningitis. *Trop. Doct.* **46**, 54–56 (2016).
118. Sudhamshu, K. C., Kumar, A. R. P., Dias, M., Shubha, A. M. & Das, K. Neurobrucellosis Infection of Ventriculoperitoneal Shunt Presenting as Peritonitis. *Indian J. Pediatr.* **83**, 1024 (2016).

**Table S1 – Search strategies**

Ovid

Database(s): EBM Reviews - Cochrane Central Register of Controlled Trials August 2022, EBM Reviews - Cochrane Database of Systematic Reviews 2005 to September 7, 2022, Embase 1974 to 2022 September 07, Ovid MEDLINE(R) and Epub Ahead of Print, In-Process, In-Data-Review & Other Non-Indexed Citations, Daily and Versions 1946 to September 07, 2022
Search Strategy:

| **#** | **Searches** | **Results** |
| --- | --- | --- |
| 1 | neurobrucellosis.ti,ab,kf. | 875 |
| 2 | exp Antibiotic Prophylaxis/ | 53102 |
| 3 | exp Anti-Bacterial Agents/ | 5048601 |
| 4 | exp antibiotic agent/ | 1662746 |
| 5 | dt.fs. | 6949513 |
| 6 | ("1 methylmocimycin*" or "11 deoxydaunorubicin*" or "14 hydroxyclarithromycin*" or "19 deformyl 4 deoxydesmycosin*" or "19 deformyldesmycosin*" or "2 acetylerythromycin*" or "2 fluoroidarubicin*" or "2 n ethylnetilmicin*" or "2 pyrrolinodoxorubicin*" or "21 aminoepothilone B" or "3 3 cyanomorpholino 3 deaminodoxorubicin*" or "3 deamino 2 fluoro 3 hydroxydoxorubicin 14 pimelate" or "3 deamino 3 morpholinodoxorubicin*" or "3 deamino 3 morpholinooxaunomycin*" or "4 demethoxy 11 deoxydaunomycinone" or "4 demethoxydaunomycinone" or "4 demethoxydoxorubicin*" or "4 deoxydesmycosin*" or "4 iodoesorubicin*" or "5 iminodaunorubicin*" or "6 n ethylnetilmicin*" or "6beta iodopenicillanic acid" or "9 deacetyl 9 methylidarubicin*" or "9 deoxydoxorubicin*" or "9 dihydroerythronolide A" or "a 102395" or "a 10255" or "a 10947" or "a 130b" or "a 192411" or "a 63075" or abkhazomycin* or abyssomicin* or acetomycin* or acetylspiramycin* or aclacinomycin* or aclarubicin* or actagardin* or actaplanin* or actinorhodine or "aculeacin A" or aculeximycin* or adicillin* or aditoprim or adriamycinone or agent* or agglomerin* or aklavinone or alafosfalin* or Alamethicin* or albocycline or aldecalmycin* or aldoxorubicin* or alisamycin* or allicin* or almecillin* or "alpha defensin*" or ambruticin* or Amdinocillin* or amfomycin* or Amikacin* or aminoglycoside* or aminopenicillin* or "Aminosalicylic Acid" or Amoxicillin* or "Amphotericin B" or Ampicillin* or amrubicin* or "angucycline derivative" or anhydrochlortetracycline or anhydroepitetracycline or anhydrotetracycline or anidulafungin* or Anisomycin* or annamycin* or ansamitocin* or "ansamycin derivative" or anthracycline* or anthracyclinone* or "anti-bacterial" or antibacterial* or "anti-bacterial*" or antibiotic* or "anti-biotic*" or antiinfective* or "anti-infective*" or antimicrobial* or "anti-microbial*" or Antimycin* or antimycobacterial* or "anti-mycobacterial*" or Antitreponemal* or "Anti-treponemal*" or Antitubercular* or "Anti-tubercular*" or apalcillin* or aplasmomycin* or "aplysianin E" or apramycin* or aristeromycin* or Arsphenamine or aspoxicillin* or astromicin* or asukamycin* or "atpenin B" or auricularum or Aurodox or aurograb or avibactam or avilamycin* or avoparcin* or azidamfenicol or azidocillin* or Azithromycin* or Azlocillin* or Aztreonam or "aztreonam lysine" or azurocidin* or bacampicillin* or Bacitracin* or bacmecillinam or bactenecin* or bacteriocid* or Bacteriocin* or bafilomycin* or balhimycin* or "baliz 2" or Bambermycin* or baquiloprim or "barminomycin I" or baycuten or beauvericin* or beroline or berubicin* or berythromycin* or "beta defensin*" or betafectin* or "beta-Lactam*" or "betaLactamase Inhibitor*" or "beta-Lactamase Inhibitor*" or betamipron or bialaphos or biapenem or bicozamycin* or "biphenomycin A" or bluensomycin* or bombinin* or "Bongkrekic Acid" or boromycin* or borrelidin* or "Brefeldin A" or brilacidin* or brobactam or butalactin* or butirosin* or cadazolid or Calcimycin* or Candicidin* or Capreomycin* or carbacephem or carbadox or carbapenem or "carbazomycin A" or Carbenicillin* or carbomycin* or Carfecillin* or carindacillin* or carubicin* or carumonam or caspofungin* or cathelicidin* or cecropin* or cefacetrile or Cefaclor or Cefadroxil or cefalexin* or cefaloglycin* or cefaloram or cefaloridine or cefalotin* or Cefamandole or cefapirin* or Cefatrizine or cefazaflur or cefazedone or Cefazolin* or cefbuperazone or cefcanel or cefcapene or cefclidin* or cefdaloxime or cefdinir or cefditoren or cefepime or cefetamet or cefetecol or cefilavancin* or Cefixime or cefluprenam or cefmatilen or Cefmenoxime or Cefmetazole or cefminox or cefodizime or Cefonicid or Cefoperazone or ceforanide or cefoselis or Cefotaxime or Cefotetan or Cefotiam or cefovecin* or Cefoxitin* or cefozopran or cefpimizole or cefpiramide or cefpirome or cefpodoxime or cefprozil or cefquinome or cefradine or cefroxadine or Cefsulodin* or ceftaroline or Ceftazidime or cefteram or ceftezole or ceftibuten or ceftiofur or Ceftizoxime or ceftobiprole or ceftolozane or Ceftriaxone or Cefuroxime or cefuzonam or Cephacetrile or Cephalexin* or Cephaloglycin* or Cephaloridine or cephalosporin* or Cephalothin* or cephamycin* or Cephapirin* or Cephradine or chalcomycin* or Chloramphenicol* or chloroorienticin* or chloropolysporin* or chlorothricin* or chlorothricolide or Chlortetracycline or "chymotrypsin trypsin*" or ciadox or "cilastatin plus imipenem" or "cinerubin A" or "cinerubin B" or cinoquidox or Ciprofloxacin* or cirramycin* or Citrinin* or Clarithromycin* or "clavulanate potassium" or "Clavulanic Acid*" or Clindamycin* or clomocycline or Cloxacillin* or colicin* or colistimethate or Colistin* or "concanamycin A" or coumamidine or coumamycin* or "cp 63956" or cryptosporin* or Cyclacillin* or cycloheximide or Cycloserine or cystargin* or "cytarabine plus daunorubicin*" or cytovaricin* or dactimicin* or Dactinomycin* or dalbaheptide or dalbavancin* or dalfopristin* or "damavaricin Fc pentyl ether" or Daptomycin* or daunomycinone or daunorubicin* or daunorubicinol or "deacetoxycephalosporin C" or deacetylcefotaxime or "deacetylcephalosporin C" or dealanylalahopcin* or decaplanin* or dechloroeremomycin* or decilorubicin* or defensin* or Demeclocycline or dermaseptin* or dermcidin* or dermostatin* or desmycosin* or detorubicin* or Diarylquinoline* or Dibekacin* or Dicloxacillin* or dihydrostreptomycin* or Diketopiperazines or dimethylchlortetracycline or dioxidine or dirithromycin* or Distamycin* or "ditrisarubicin B" or doripenem or doxorubicin* or doxorubicinol or Doxycycline or drosocin* or drug* or echinocandin* or Echinomycin* or Edeine or efepristin* or efrotomycin* or emimycin* or endusamycin* or enniatin* or Enoxacin* or Enviomycin* or eperezolid or epetraborole or epicillin* or epidermin* or epiderstatin* or epiroprim or epirubicin* or epirubicinol or epitetracycline or epothilone* or "epsilon rhodomycinone" or eravacycline or eremomycin* or ertapenem or Erythromycin* or erythromycylamine or erythronolide* or esorubicin* or Ethambutol or Ethionamide or ethylhydrocupreine or etimicin* or evernimicin* or everninomicin* or faeriefungin* or fidaxomicin* or Filipin* or "fleroxacin deacetylcefotaxime ester" or flomoxef or flopristin* or florfenicol or Floxacillin* or flucloxacillin* or flumoxil or Fluoroquinolone* or flurithromycin* or fomidacillin* or fortimicin* or Fosfomycin* or fosmidomycin* or Framycetin* or fropenem or fungichromin* or furaquinocin* or furazidin* or "furazolium chloride" or furbenicillin* or fusafungine or "fusidate sodium" or "Fusidic Acid" or fuzlocillin* or galarubicin* or gallidermin* or gamithromycin* or ganefromycin* or "ge 2270a" or gentamicin* or gepotidacin* or globomycin* or gloximonam or "glycylcycline derivative" or "gonadorelin6 dextro lysine 2 pyrrolinodoxorubicin*" or "goniodomin A" or Gramicidin* or granulysin* or grisein* or guamecycline or habekacin* or hamycin* or hatomamicin* or hedamycin* or heliomycin* or hepcidin* or hetacillin* or hexacycline or hidamicin* or "histatin 5" or histatin* or hygromycin* or hymeglusin* or hypothemycin* or iclaprim or idarubicin* or idarubicinol or ikarugamycin* or "imidacloprid plus moxidectin*" or Imipenem or indolicidin* or inostamycin* or intervention* or iseganan or isepamicin* or Isoniazid or "isopenicillin N" or "isoswinholide A" or istamycin* or "iturin A" or ixabepilone or Josamycin* or "k 252a" or kalafungin* or Kanamycin* or kanendomycin* or kasugamycin* or kelfiprim or ketolide or kidamycin* or kinamycin* or Kitasamycin* or "l 156602" or "l 733560" or "l 786392" or lactacystin* or Lactams or lactivicin* or "lactocin S" or lactococcin* or lactoferricin* or ladirubicin* or laidlomycin* or lancovutide or lankamycin* or lanopepden or lanthiopeptin* or lantibiotic or Lasalocid or latamoxef or lavanducyanin* or lefamulin* or lenampicillin* or lenapenem or lenoremycin* or Leprostatic* or "leucinostatin A" or "leucinostatin B" or Leucomycin* or leurubicin* or Levofloxacin* or lexithromycin* or "lff 571" or Lincomycin* or lincosamide* or Linezolid or linopristin* or lividomycin* or "lonomycin A" or loracarbef or lotilibcin* or Lucensomycin* or lydicamycin* or Lymecycline or lysobactin* or lysocellin* or lysostaphin* or macrolide or magainin* or malyngolide or manag* or manumycin* or maridomycin* or "mdl 62208" or "mdl 62211" or mecillinam or meclocycline or medication* or megacin* or megalomicin* or Mepartricin* or meropenem or mersacidin* or metacycline or metampicillin* or Methacycline or Methicillin* or "methylenomycin A" or "methylenomycin B" or methymycin* or methynolide or meticillin* or Mezlocillin* or microbicid* or "microcin b17" or "microcin J25" or micronomicin* or midecamycin* or mideplanin* or "mikamycin B" or Mikamycin* or "milbemycin oxime" or milbemycin* or Minocycline or Miocamycin* or miokamycin* or miporamicin* or miraxid or mocimycin* or "moenomycin A" or monensin* or "monobactam derivative" or Moxalactam or moxidectin* or "ms 8209" or Mupirocin* or mureidomycin* or murepavadin* or mycinamicin* or Mycobacillin* or mycolog or mycoticin* or myxothiazol or "n benzyldoxorubicin 14 valerate" or "n trifluoroacetyldoxorubicin*" or Nafcillin* or "Nalidixic Acid" or narasin* or Natamycin* or neamine or nebacetin* or Nebramycin* or negamycin* or nemadectin* or nemorubicin* or Neomycin* or neosporin* or Netilmicin* or Netropsin* or niddamycin* or Nigericin* or Nisin* or nitrocefin* or nitrosochloramphenicol or "nocardicin A" or "nocardicin E" or "nocardicinic acid derivative*" or Norfloxacin* or nosiheptide or nourseothricin* or Novobiocin* or "nvb 302" or nybomycin* or Nystatin* or "oasomycin A" or obelmycin* or Ofloxacin* or olaquindox or oleandolide or Oleandomycin* or oligomycin* or omadacycline or omiganan or optocillin* or "orienticin A" or orientiparcin* or oritavancin* or oropivalone or Oxacillin* or oxaunomycin* or "Oxolinic Acid" or Oxytetracycline or paldimycin* or panipenem or pardaxin* or Paromomycin* or patulin* or pediazole or pediocin* or Pefloxacin* or penamecillin* or penethamate or "Penicillanic Acid" or "Penicillic Acid" or penicillin* or "penicilloic acid" or pentalenolactone or pentisomicin* or peptaibol or pexiganan or "pf 708093" or pharmacotherap* or phenelfamycin* or pheneticillin* or phleomycin* or pikromycin* or "Pipemidic Acid" or Piperacillin* or pirarubicin* or pirazmonam or pirlimycin* or Pivampicillin* or pivmecillinam or platensimycin* or plazomicin* or plectasin* or pleuromutilin* or pluramycin* or pneumocandin* or "polyactin A" or polyfungin* or polymyxin* or "polyoxin B" or "polyoxin D" or polysporin* or polytrim or posizolid or "pr 39" or Pristinamycin* or Prodigiosin* or prohepcidin* or propicillin* or protegrin* or Prothionamide or prothracarcin* or "pseudomonic acid" or Pyrazinamide or pyrromycinone or pyrroxamycin* or quinacillin* or quinomycin* or quinupristin* or radezolid or radicicol or ramoplanin* or ranalexin* or ranbezolid or razupenem or retacillin* or retapamulin* or "rhodomycin A" or Ribostamycin* or Rifabutin* or Rifampin* or Rifamycin* or rimocidin* or Ristocetin* or ritipenem or "ritipenem acoxil" or rodorubicin* or roflamycoin* or rokitamycin* or Rolitetracycline or rosaramicin* or Roxarsone or Roxithromycin* or ruboxyl or Rutamycin* or sabarubicin* or sagopilone or sanfetrinem or sarecycline or "simaomicin alpha" or "simocyclinone D8" or Sirolimus or "sisomicin sulfate" or Sisomicin* or "skf 104662" or sofradex or Spectinomycin* or "spinosyn A" or Spiramycin* or squalamine or stigmatellin* or streptoduocin* or Streptogramin* or streptolydigin* or Streptomycin* or streptothricin* or streptotriad or Streptovaricin* or streptovirudin* or "streptovitacin A" or stubomycin* or subtilin* or Sulbactam or Sulbenicillin* or Sulfamerazine or Sulfamethoxypyridazine or sulfazecin* or sulopenem or sultamicillin* or surfactin* or surotomycin* or "swinholide A" or "swinholide B" or tachyplesin* or Talampicillin* or tameticillin* or tazobactam or tebipenem or tedizolid or Teicoplanin* or teixobactin* or telavancin* or temocillin* or terdecamycin* or tetracyclin* or Tetracycline or tetramycin* or tetronasin* or tetronomycin* or tetroxoprim or therap* or Thiamphenicol or Thienamycin* or Thioacetazone or thiolactomycin* or "thionin peptide" or thiopeptin* or thiophenoxycefalotin* or Thiostrepton or tiamulin* or "tibezonium iodide" or Ticarcillin* or tigecycline or tigemonam or tildipirosin* or tilmicosin* or timentin* or tirandamycin* or tizoxanide or tobicillin* or Tobramycin* or tolramycin* or tomopenem or toyocamycin* or treatment* or tresaderm or tribactam or trichomycin* or "trichostatic acid" or "trichostatin A" or trimethoprim* or "trinem derivative" or "triostin A" or triplopen or trisep or Troleandomycin* or trospectomycin* or tuftsin* or tulathromycin* or Tunicamycin* or tutofusin* or Tylosin* or tylvalosin* or Tyrocidine or Tyrothricin* or "u 78608" or "uk 69753" or unphenelfamycin* or "urdamycin C" or "urdamycin D" or "urdamycin H" or ureidopenicillin* or urobiotic or "vacidin A" or validamycin* or Valinomycin* or valnemulin* or valrubicin* or Vancomycin* or venturicidin* or vernamycin* or "violamycin B1" or Viomycin* or "virginiae butanolide A" or "virginiae butanolide C" or "virginiamycin M" or "virginiamycin S" or Virginiamycin* or "viriplanin A" or viscosin* or volpristin* or "ws 9659 b" or "zibrofusidic acid" or zineryt or "zoptarelin doxorubicin*" or zorbamycin* or zorubicin*).ti,ab,kf. | 25562213 |
| 7 | or/2-6 | 28708188 |
| 8 | 1 and 7 | 623 |
| 9 | (exp animals/ or exp nonhuman/) not exp humans/ | 11923418 |
| 10 | ((alpaca or alpacas or amphibian or amphibians or animal or animals or antelope or armadillo or armadillos or avian or baboon or baboons or beagle or beagles or bee or bees or bird or birds or bison or bovine or buffalo or buffaloes or buffalos or "c elegans" or "Caenorhabditis elegans" or camel or camels or canine or canines or carp or cats or cattle or chick or chicken or chickens or chicks or chimp or chimpanze or chimpanzees or chimps or cow or cows or "D melanogaster" or "dairy calf" or "dairy calves" or deer or dog or dogs or donkey or donkeys or drosophila or "Drosophila melanogaster" or duck or duckling or ducklings or ducks or equid or equids or equine or equines or feline or felines or ferret or ferrets or finch or finches or fish or flatworm or flatworms or fox or foxes or frog or frogs or "fruit flies" or "fruit fly" or "G mellonella" or "Galleria mellonella" or geese or gerbil or gerbils or goat or goats or goose or gorilla or gorillas or hamster or hamsters or hare or hares or heifer or heifers or horse or horses or insect or insects or jellyfish or kangaroo or kangaroos or kitten or kittens or lagomorph or lagomorphs or lamb or lambs or lemur or lemurs or llama or llamas or macaque or macaques or macaw or macaws or marmoset or marmosets or mice or minipig or minipigs or mink or minks or monkey or monkeys or mouse or mule or mules or nematode or nematodes or octopus or octopuses or orangutan or "orang-utan" or orangutans or "orang-utans" or ostrich or ostriches or oxen or parrot or parrots or pig or pigeon or pigeons or piglet or piglets or pigs or porcine or primate or primates or quail or rabbit or rabbits or rat or rats or reptile or reptiles or rodent or rodents or ruminant or ruminants or salmon or sheep or shrimp or slug or slugs or swine or tamarin or tamarins or toad or toads or trout or urchin or urchins or vole or voles or waxworm or waxworms or wildlife or worm or worms or xenopus or "zebra fish" or zebrafish) not (human or humans or patient or patients)).ti,ab,hw,kf. | 10184103 |
| 11 | 8 not (9 or 10) | 614 |
| 12 | limit 11 to (editorial or erratum or note or addresses or autobiography or bibliography or biography or blogs or comment or dictionary or directory or interactive tutorial or interview or lectures or legal cases or legislation or news or newspaper article or overall or patient education handout or periodical index or portraits or published erratum or video-audio media or webcasts) [Limit not valid in CCTR,CDSR,Embase,Ovid MEDLINE(R),Ovid MEDLINE(R) Daily Update,Ovid MEDLINE(R) PubMed not MEDLINE,Ovid MEDLINE(R) In-Process,Ovid MEDLINE(R) Publisher; records were retained] | 4 |
| 13 | 11 not 12 | 610 |
| 14 | remove duplicates from 13 | 411 |

Scopus

1 TITLE-ABS-KEY(neurobrucellosis)

2 TITLE-ABS-KEY("1 methylmocimycin*" OR "11 deoxydaunorubicin*" OR "14 hydroxyclarithromycin*" OR "19 deformyl 4 deoxydesmycosin*" OR "19 deformyldesmycosin*" OR "2 acetylerythromycin*" OR "2 fluoroidarubicin*" OR "2 n ethylnetilmicin*" OR "2 pyrrolinodoxorubicin*" OR "21 aminoepothilone B" OR "3 3 cyanomorpholino 3 deaminodoxorubicin*" OR "3 deamino 2 fluoro 3 hydroxydoxorubicin 14 pimelate" OR "3 deamino 3 morpholinodoxorubicin*" OR "3 deamino 3 morpholinooxaunomycin*" OR "4 demethoxy 11 deoxydaunomycinone" OR "4 demethoxydaunomycinone" OR "4 demethoxydoxorubicin*" OR "4 deoxydesmycosin*" OR "4 iodoesorubicin*" OR "5 iminodaunorubicin*" OR "6 n ethylnetilmicin*" OR "6beta iodopenicillanic acid" OR "9 deacetyl 9 methylidarubicin*" OR "9 deoxydoxorubicin*" OR "9 dihydroerythronolide A" OR "a 102395" OR "a 10255" OR "a 10947" OR "a 130b" OR "a 192411" OR "a 63075" OR abkhazomycin* OR abyssomicin* OR acetomycin* OR acetylspiramycin* OR aclacinomycin* OR aclarubicin* OR actagardin* OR actaplanin* OR actinorhodine OR "aculeacin A" OR aculeximycin* OR adicillin* OR aditoprim OR adriamycinone OR agent* OR agglomerin* OR aklavinone OR alafosfalin* OR Alamethicin* OR albocycline OR aldecalmycin* OR aldoxorubicin* OR alisamycin* OR allicin* OR almecillin* OR "alpha defensin*" OR ambruticin* OR Amdinocillin* OR amfomycin* OR Amikacin* OR aminoglycoside* OR aminopenicillin* OR "Aminosalicylic Acid" OR Amoxicillin* OR "Amphotericin B" OR Ampicillin* OR amrubicin* OR "angucycline derivative" OR anhydrochlortetracycline OR anhydroepitetracycline OR anhydrotetracycline OR anidulafungin* OR Anisomycin* OR annamycin* OR ansamitocin* OR "ansamycin derivative" OR anthracycline* OR anthracyclinone* OR "anti-bacterial" OR antibacterial* OR "anti-bacterial*" OR antibiotic* OR "anti-biotic*" OR antiinfective* OR "anti-infective*" OR antimicrobial* OR "anti-microbial*" OR Antimycin* OR antimycobacterial* OR "anti-mycobacterial*" OR Antitreponemal* OR "Anti-treponemal*" OR Antitubercular* OR "Anti-tubercular*" OR apalcillin* OR aplasmomycin* OR "aplysianin E" OR apramycin* OR aristeromycin* OR Arsphenamine OR aspoxicillin* OR astromicin* OR asukamycin* OR "atpenin B" OR auricularum OR Aurodox OR aurograb OR avibactam OR avilamycin* OR avoparcin* OR azidamfenicol OR azidocillin* OR Azithromycin* OR Azlocillin* OR Aztreonam OR "aztreonam lysine" OR azurocidin* OR bacampicillin* OR Bacitracin* OR bacmecillinam OR bactenecin* OR bacteriocid* OR Bacteriocin* OR bafilomycin* OR balhimycin* OR "baliz 2" OR Bambermycin* OR baquiloprim OR "barminomycin I" OR baycuten OR beauvericin* OR beroline OR berubicin* OR berythromycin* OR "beta defensin*" OR betafectin* OR "beta-Lactam*" OR "betaLactamase Inhibitor*" OR "beta-Lactamase Inhibitor*" OR betamipron OR bialaphos OR biapenem OR bicozamycin* OR "biphenomycin A" OR bluensomycin* OR bombinin* OR "Bongkrekic Acid" OR boromycin* OR borrelidin* OR "Brefeldin A" OR brilacidin* OR brobactam OR butalactin* OR butirosin* OR cadazolid OR Calcimycin* OR Candicidin* OR Capreomycin* OR carbacephem OR carbadox OR carbapenem OR "carbazomycin A" OR Carbenicillin* OR carbomycin* OR Carfecillin* OR carindacillin* OR carubicin* OR carumonam OR caspofungin* OR cathelicidin* OR cecropin* OR cefacetrile OR Cefaclor OR Cefadroxil OR cefalexin* OR cefaloglycin* OR cefaloram OR cefaloridine OR cefalotin* OR Cefamandole OR cefapirin* OR Cefatrizine OR cefazaflur OR cefazedone OR Cefazolin* OR cefbuperazone OR cefcanel OR cefcapene OR cefclidin* OR cefdaloxime OR cefdinir OR cefditoren OR cefepime OR cefetamet OR cefetecol OR cefilavancin* OR Cefixime OR cefluprenam OR cefmatilen OR Cefmenoxime OR Cefmetazole OR cefminox OR cefodizime OR Cefonicid OR Cefoperazone OR ceforanide OR cefoselis OR Cefotaxime OR Cefotetan OR Cefotiam OR cefovecin* OR Cefoxitin* OR cefozopran OR cefpimizole OR cefpiramide OR cefpirome OR cefpodoxime OR cefprozil OR cefquinome OR cefradine OR cefroxadine OR Cefsulodin* OR ceftaroline OR Ceftazidime OR cefteram OR ceftezole OR ceftibuten OR ceftiofur OR Ceftizoxime OR ceftobiprole OR ceftolozane OR Ceftriaxone OR Cefuroxime OR cefuzonam OR Cephacetrile OR Cephalexin* OR Cephaloglycin* OR Cephaloridine OR cephalosporin* OR Cephalothin* OR cephamycin* OR Cephapirin* OR Cephradine OR chalcomycin* OR Chloramphenicol* OR chloroorienticin* OR chloropolysporin* OR chlorothricin* OR chlorothricolide OR Chlortetracycline OR "chymotrypsin trypsin*" OR ciadox OR "cilastatin plus imipenem" OR "cinerubin A" OR "cinerubin B" OR cinoquidox OR Ciprofloxacin* OR cirramycin* OR Citrinin* OR Clarithromycin* OR "clavulanate potassium" OR "Clavulanic Acid*" OR Clindamycin* OR clomocycline OR Cloxacillin* OR colicin* OR colistimethate OR Colistin* OR "concanamycin A" OR coumamidine OR coumamycin* OR "cp 63956" OR cryptosporin* OR Cyclacillin* OR cycloheximide OR Cycloserine OR cystargin* OR "cytarabine plus daunorubicin*" OR cytovaricin* OR dactimicin* OR Dactinomycin* OR dalbaheptide OR dalbavancin* OR dalfopristin* OR "damavaricin Fc pentyl ether" OR Daptomycin* OR daunomycinone OR daunorubicin* OR daunorubicinol OR "deacetoxycephalosporin C" OR deacetylcefotaxime OR "deacetylcephalosporin C" OR dealanylalahopcin* OR decaplanin* OR dechloroeremomycin* OR decilorubicin* OR defensin* OR Demeclocycline OR dermaseptin* OR dermcidin* OR dermostatin* OR desmycosin* OR detorubicin* OR Diarylquinoline* OR Dibekacin* OR Dicloxacillin* OR dihydrostreptomycin* OR Diketopiperazines OR dimethylchlortetracycline OR dioxidine OR dirithromycin* OR Distamycin* OR "ditrisarubicin B" OR doripenem OR doxorubicin* OR doxorubicinol OR Doxycycline OR drosocin* OR drug* OR echinocandin* OR Echinomycin* OR Edeine OR efepristin* OR efrotomycin* OR emimycin* OR endusamycin* OR enniatin* OR Enoxacin* OR Enviomycin* OR eperezolid OR epetraborole OR epicillin* OR epidermin* OR epiderstatin* OR epiroprim OR epirubicin* OR epirubicinol OR epitetracycline OR epothilone* OR "epsilon rhodomycinone" OR eravacycline OR eremomycin* OR ertapenem OR Erythromycin* OR erythromycylamine OR erythronolide* OR esorubicin* OR Ethambutol OR Ethionamide OR ethylhydrocupreine OR etimicin* OR evernimicin* OR everninomicin* OR faeriefungin* OR fidaxomicin* OR Filipin* OR "fleroxacin deacetylcefotaxime ester" OR flomoxef OR flopristin* OR florfenicol OR Floxacillin* OR flucloxacillin* OR flumoxil OR Fluoroquinolone* OR flurithromycin* OR fomidacillin* OR fortimicin* OR Fosfomycin* OR fosmidomycin* OR Framycetin* OR fropenem OR fungichromin* OR furaquinocin* OR furazidin* OR "furazolium chloride" OR furbenicillin* OR fusafungine OR "fusidate sodium" OR "Fusidic Acid" OR fuzlocillin* OR galarubicin* OR gallidermin* OR gamithromycin* OR ganefromycin* OR "ge 2270a" OR gentamicin* OR gepotidacin* OR globomycin* OR gloximonam OR "glycylcycline derivative" OR "gonadorelin6 dextro lysine 2 pyrrolinodoxorubicin*" OR "goniodomin A" OR Gramicidin* OR granulysin* OR grisein* OR guamecycline OR habekacin* OR hamycin* OR hatomamicin* OR hedamycin* OR heliomycin* OR hepcidin* OR hetacillin* OR hexacycline OR hidamicin* OR "histatin 5" OR histatin* OR hygromycin* OR hymeglusin* OR hypothemycin* OR iclaprim OR idarubicin* OR idarubicinol OR ikarugamycin* OR "imidacloprid plus moxidectin*" OR Imipenem OR indolicidin* OR inostamycin* OR intervention* OR iseganan OR isepamicin* OR Isoniazid OR "isopenicillin N" OR "isoswinholide A" OR istamycin* OR "iturin A" OR ixabepilone OR Josamycin* OR "k 252a" OR kalafungin* OR Kanamycin* OR kanendomycin* OR kasugamycin* OR kelfiprim OR ketolide OR kidamycin* OR kinamycin* OR Kitasamycin* OR "l 156602" OR "l 733560" OR "l 786392" OR lactacystin* OR Lactams OR lactivicin* OR "lactocin S" OR lactococcin* OR lactoferricin* OR ladirubicin* OR laidlomycin* OR lancovutide OR lankamycin* OR lanopepden OR lanthiopeptin* OR lantibiotic OR Lasalocid OR latamoxef OR lavanducyanin* OR lefamulin* OR lenampicillin* OR lenapenem OR lenoremycin* OR Leprostatic* OR "leucinostatin A" OR "leucinostatin B" OR Leucomycin* OR leurubicin* OR Levofloxacin* OR lexithromycin* OR "lff 571" OR Lincomycin* OR lincosamide* OR Linezolid OR linopristin* OR lividomycin* OR "lonomycin A" OR loracarbef OR lotilibcin* OR Lucensomycin* OR lydicamycin* OR Lymecycline OR lysobactin* OR lysocellin* OR lysostaphin* OR macrolide OR magainin* OR malyngolide OR manag* OR manumycin* OR maridomycin* OR "mdl 62208" OR "mdl 62211" OR mecillinam OR meclocycline OR medication* OR megacin* OR megalomicin* OR Mepartricin* OR meropenem OR mersacidin* OR metacycline OR metampicillin* OR Methacycline OR Methicillin* OR "methylenomycin A" OR "methylenomycin B" OR methymycin* OR methynolide OR meticillin* OR Mezlocillin* OR microbicid* OR "microcin b17" OR "microcin J25" OR micronomicin* OR midecamycin* OR mideplanin* OR "mikamycin B" OR Mikamycin* OR "milbemycin oxime" OR milbemycin* OR Minocycline OR Miocamycin* OR miokamycin* OR miporamicin* OR miraxid OR mocimycin* OR "moenomycin A" OR monensin* OR "monobactam derivative" OR Moxalactam OR moxidectin* OR "ms 8209" OR Mupirocin* OR mureidomycin* OR murepavadin* OR mycinamicin* OR Mycobacillin* OR mycolog OR mycoticin* OR myxothiazol OR "n benzyldoxorubicin 14 valerate" OR "n trifluoroacetyldoxorubicin*" OR Nafcillin* OR "Nalidixic Acid" OR narasin* OR Natamycin* OR neamine OR nebacetin* OR Nebramycin* OR negamycin* OR nemadectin* OR nemorubicin* OR Neomycin* OR neosporin* OR Netilmicin* OR Netropsin* OR niddamycin* OR Nigericin* OR Nisin* OR nitrocefin* OR nitrosochloramphenicol OR "nocardicin A" OR "nocardicin E" OR "nocardicinic acid derivative*" OR Norfloxacin* OR nosiheptide OR nourseothricin* OR Novobiocin* OR "nvb 302" OR nybomycin* OR Nystatin* OR "oasomycin A" OR obelmycin* OR Ofloxacin* OR olaquindox OR oleandolide OR Oleandomycin* OR oligomycin* OR omadacycline OR omiganan OR optocillin* OR "orienticin A" OR orientiparcin* OR oritavancin* OR oropivalone OR Oxacillin* OR oxaunomycin* OR "Oxolinic Acid" OR Oxytetracycline OR paldimycin* OR panipenem OR pardaxin* OR Paromomycin* OR patulin* OR pediazole OR pediocin* OR Pefloxacin* OR penamecillin* OR penethamate OR "Penicillanic Acid" OR "Penicillic Acid" OR penicillin* OR "penicilloic acid" OR pentalenolactone OR pentisomicin* OR peptaibol OR pexiganan OR "pf 708093" OR pharmacotherap* OR phenelfamycin* OR pheneticillin* OR phleomycin* OR pikromycin* OR "Pipemidic Acid" OR Piperacillin* OR pirarubicin* OR pirazmonam OR pirlimycin* OR Pivampicillin* OR pivmecillinam OR platensimycin* OR plazomicin* OR plectasin* OR pleuromutilin* OR pluramycin* OR pneumocandin* OR "polyactin A" OR polyfungin* OR polymyxin* OR "polyoxin B" OR "polyoxin D" OR polysporin* OR polytrim OR posizolid OR "pr 39" OR Pristinamycin* OR Prodigiosin* OR prohepcidin* OR propicillin* OR protegrin* OR Prothionamide OR prothracarcin* OR "pseudomonic acid" OR Pyrazinamide OR pyrromycinone OR pyrroxamycin* OR quinacillin* OR quinomycin* OR quinupristin* OR radezolid OR radicicol OR ramoplanin* OR ranalexin* OR ranbezolid OR razupenem OR retacillin* OR retapamulin* OR "rhodomycin A" OR Ribostamycin* OR Rifabutin* OR Rifampin* OR Rifamycin* OR rimocidin* OR Ristocetin* OR ritipenem OR "ritipenem acoxil" OR rodorubicin* OR roflamycoin* OR rokitamycin* OR Rolitetracycline OR rosaramicin* OR Roxarsone OR Roxithromycin* OR ruboxyl OR Rutamycin* OR sabarubicin* OR sagopilone OR sanfetrinem OR sarecycline OR "simaomicin alpha" OR "simocyclinone D8" OR Sirolimus OR "sisomicin sulfate" OR Sisomicin* OR "skf 104662" OR sofradex OR Spectinomycin* OR "spinosyn A" OR Spiramycin* OR squalamine OR stigmatellin* OR streptoduocin* OR Streptogramin* OR streptolydigin* OR Streptomycin* OR streptothricin* OR streptotriad OR Streptovaricin* OR streptovirudin* OR "streptovitacin A" OR stubomycin* OR subtilin* OR Sulbactam OR Sulbenicillin* OR Sulfamerazine OR Sulfamethoxypyridazine OR sulfazecin* OR sulopenem OR sultamicillin* OR surfactin* OR surotomycin* OR "swinholide A" OR "swinholide B" OR tachyplesin* OR Talampicillin* OR tameticillin* OR tazobactam OR tebipenem OR tedizolid OR Teicoplanin* OR teixobactin* OR telavancin* OR temocillin* OR terdecamycin* OR tetracyclin* OR Tetracycline OR tetramycin* OR tetronasin* OR tetronomycin* OR tetroxoprim OR therap* OR Thiamphenicol OR Thienamycin* OR Thioacetazone OR thiolactomycin* OR "thionin peptide" OR thiopeptin* OR thiophenoxycefalotin* OR Thiostrepton OR tiamulin* OR "tibezonium iodide" OR Ticarcillin* OR tigecycline OR tigemonam OR tildipirosin* OR tilmicosin* OR timentin* OR tirandamycin* OR tizoxanide OR tobicillin* OR Tobramycin* OR tolramycin* OR tomopenem OR toyocamycin* OR treatment* OR tresaderm OR tribactam OR trichomycin* OR "trichostatic acid" OR "trichostatin A" OR trimethoprim* OR "trinem derivative" OR "triostin A" OR triplopen OR trisep OR Troleandomycin* OR trospectomycin* OR tuftsin* OR tulathromycin* OR Tunicamycin* OR tutofusin* OR Tylosin* OR tylvalosin* OR Tyrocidine OR Tyrothricin* OR "u 78608" OR "uk 69753" OR unphenelfamycin* OR "urdamycin C" OR "urdamycin D" OR "urdamycin H" OR ureidopenicillin* OR urobiotic OR "vacidin A" OR validamycin* OR Valinomycin* OR valnemulin* OR valrubicin* OR Vancomycin* OR venturicidin* OR vernamycin* OR "violamycin B1" OR Viomycin* OR "virginiae butanolide A" OR "virginiae butanolide C" OR "virginiamycin M" OR "virginiamycin S" OR Virginiamycin* OR "viriplanin A" OR viscosin* OR volpristin* OR "ws 9659 b" OR "zibrofusidic acid" OR zineryt OR "zoptarelin doxorubicin*" OR zorbamycin* OR zorubicin*)

3 1 and 2

4 TITLE-ABS-KEY((alpaca OR alpacas OR amphibian OR amphibians OR animal OR animals OR antelope OR armadillo OR armadillos OR avian OR baboon OR baboons OR beagle OR beagles OR bee OR bees OR bird OR birds OR bison OR bovine OR buffalo OR buffaloes OR buffalos OR "c elegans" OR "Caenorhabditis elegans" OR camel OR camels OR canine OR canines OR carp OR cats OR cattle OR chick OR chicken OR chickens OR chicks OR chimp OR chimpanze OR chimpanzees OR chimps OR cow OR cows OR "D melanogaster" OR "dairy calf" OR "dairy calves" OR deer OR dog OR dogs OR donkey OR donkeys OR drosophila OR "Drosophila melanogaster" OR duck OR duckling OR ducklings OR ducks OR equid OR equids OR equine OR equines OR feline OR felines OR ferret OR ferrets OR finch OR finches OR fish OR flatworm OR flatworms OR fox OR foxes OR frog OR frogs OR "fruit flies" OR "fruit fly" OR "G mellonella" OR "Galleria mellonella" OR geese OR gerbil OR gerbils OR goat OR goats OR goose OR gorilla OR gorillas OR hamster OR hamsters OR hare OR hares OR heifer OR heifers OR horse OR horses OR insect OR insects OR jellyfish OR kangaroo OR kangaroos OR kitten OR kittens OR lagomorph OR lagomorphs OR lamb OR lambs OR llama OR llamas OR macaque OR macaques OR macaw OR macaws OR marmoset OR marmosets OR mice OR minipig OR minipigs OR mink OR minks OR monkey OR monkeys OR mouse OR mule OR mules OR nematode OR nematodes OR octopus OR octopuses OR orangutan OR "orang-utan" OR orangutans OR "orang-utans" OR oxen OR parrot OR parrots OR pig OR pigeon OR pigeons OR piglet OR piglets OR pigs OR porcine OR primate OR primates OR quail OR rabbit OR rabbits OR rat OR rats OR reptile OR reptiles OR rodent OR rodents OR ruminant OR ruminants OR salmon OR sheep OR shrimp OR slug OR slugs OR swine OR tamarin OR tamarins OR toad OR toads OR trout OR urchin OR urchins OR vole OR voles OR waxworm OR waxworms OR worm OR worms OR xenopus OR "zebra fish" OR zebrafish) AND NOT (human OR humans or patient or patients))

5 3 and not 4

6 DOCTYPE(ed) OR DOCTYPE(bk) OR DOCTYPE(er) OR DOCTYPE(no) OR DOCTYPE(sh)

7 5 and not 6

8 INDEX(embase) OR INDEX(medline) OR PMID(0* OR 1* OR 2* OR 3* OR 4* OR 5* OR 6* OR 7* OR 8* OR 9*)

9 7 and not 8

**Table S2**– Cerebrospinal fluid parameters during patient’s treatment

|  | WBCs (cells/uL) | Glucose (mg/dl) | Protein (mg/dL) |
| --- | --- | --- | --- |
| 05-May-21 | 5 19 | 25 | 277 |
| 25-May-21 | 271 | 35 | 215 |
| 17-Jun-21 | 117 | 29 | 188 |
| 01-Jul-21 | 91 | 35 | 154 |
| 29-Jul-21 | 93 | 33 | 104 |
| 19-Nov-21 | 19 | 47 | 64 |
| 19-May- 22 | 16 | 54 | 53 |
| 08-Jun-23 | 9 | 53 | 37 |

WBCs: white blood cells.

# **Table S3** – Multivariable Analysis assessing assessing factors associated with a poor recovery status considering the duration of treatment as a categorical variable (>6 months)

| Variable | Recovery  aOR (95% CI) | P value |
| --- | --- | --- |
| Age | 0.99 (0.97-1.01) | 0.37 |
| Treatment duration (> 6 months) | 0.8 (0.38-1.69) | 0.55 |
| Use of ceftriaxone | 0.97 (0.46-2.05) | 0.94 |
| Use of corticosteroids | 0.4 (0.16-0.99) | 0.045 |
| Motor deficit | 0.31 (0.14-0.65) | 0.002 |
| Hearing loss | 0.04 (0.01-0.11) | <0.001 |

aOR=adjusted odds ratio; CI=confidence interval

# **Table S4** – Multivariable secondary analysis assessing factors associated with a poor recovery status considering the onset of symptoms as a categorical variable (> 3 months).

| Variable | Recovery  aOR (95% CI) | P value |
| --- | --- | --- |
| Age | 0.99 (0.97-1.0) | 0.70 |
| Treatment duration | 0.99 (0.86-1.13) | 0.85 |
| Use of ceftriaxone | 0.85 (0.35-2.06) | 0.71 |
| Use of corticosteroids | 0.26 (0.1-0.71) | 0.009 |
| Motor deficit | 0.53 (0.22-1.29) | 0.16 |
| Hearing loss | 0.06 (0.02-0.2) | <0.001 |
| Onset of symptoms > 3 months | 0.63 (0.24-1.67) | 0.34 |

aOR=adjusted odds ratio; CI=confidence interval

Table S5 – PRISMA 2020 Checklist

| **Section and Topic** | **Item #** | **Checklist item** | **Location where item is reported** |
| --- | --- | --- | --- |
| **TITLE** | | |  |
| Title | 1 | Identify the report as a systematic review. | Lines 1-3 |
| **ABSTRACT** | | |  |
| Abstract | 2 | See the PRISMA 2020 for Abstracts checklist. | Lines 27-37 |
| **INTRODUCTION** | | |  |
| Rationale | 3 | Describe the rationale for the review in the context of existing knowledge. | Lines 62-64 |
| Objectives | 4 | Provide an explicit statement of the objective(s) or question(s) the review addresses. | Lines 65-69 |
| **METHODS** | | |  |
| Eligibility criteria | 5 | Specify the inclusion and exclusion criteria for the review and how studies were grouped for the syntheses. | Lines 90-96 |
| Information sources | 6 | Specify all databases, registers, websites, organisations, reference lists and other sources searched or consulted to identify studies. Specify the date when each source was last searched or consulted. | Lines 81-84 |
| Search strategy | 7 | Present the full search strategies for all databases, registers and websites, including any filters and limits used. | Table S1 |
| Selection process | 8 | Specify the methods used to decide whether a study met the inclusion criteria of the review, including how many reviewers screened each record and each report retrieved, whether they worked independently, and if applicable, details of automation tools used in the process. | Lines 97-99 |
| Data collection process | 9 | Specify the methods used to collect data from reports, including how many reviewers collected data from each report, whether they worked independently, any processes for obtaining or confirming data from study investigators, and if applicable, details of automation tools used in the process. | Lines 104-106 |
| Data items | 10a | List and define all outcomes for which data were sought. Specify whether all results that were compatible with each outcome domain in each study were sought (e.g. for all measures, time points, analyses), and if not, the methods used to decide which results to collect. | Lines 137-140 |
|  | 10b | List and define all other variables for which data were sought (e.g. participant and intervention characteristics, funding sources). Describe any assumptions made about any missing or unclear information. | Not present |
| Study risk of bias assessment | 11 | Specify the methods used to assess risk of bias in the included studies, including details of the tool(s) used, how many reviewers assessed each study and whether they worked independently, and if applicable, details of automation tools used in the process. | Lines 100-101 |
| Effect measures | 12 | Specify for each outcome the effect measure(s) (e.g. risk ratio, mean difference) used in the synthesis or presentation of results. | Line 150 |
| Synthesis methods | 13a | Describe the processes used to decide which studies were eligible for each synthesis (e.g. tabulating the study intervention characteristics and comparing against the planned groups for each synthesis (item #5)). | Not applicable |
|  | 13b | Describe any methods required to prepare the data for presentation or synthesis, such as handling of missing summary statistics, or data conversions. | Not applicable |
|  | 13c | Describe any methods used to tabulate or visually display results of individual studies and syntheses. | Not applicable |
|  | 13d | Describe any methods used to synthesize results and provide a rationale for the choice(s). If meta-analysis was performed, describe the model(s), method(s) to identify the presence and extent of statistical heterogeneity, and software package(s) used. | Lines 143-156 |
|  | 13e | Describe any methods used to explore possible causes of heterogeneity among study results (e.g. subgroup analysis, meta-regression). | Not applicable |
|  | 13f | Describe any sensitivity analyses conducted to assess robustness of the synthesized results. | Lines 157-158 |
| Reporting bias assessment | 14 | Describe any methods used to assess risk of bias due to missing results in a synthesis (arising from reporting biases). | Not applicable |
| Certainty assessment | 15 | Describe any methods used to assess certainty (or confidence) in the body of evidence for an outcome. | Line 159 |
| **RESULTS** | | |  |
| Study selection | 16a | Describe the results of the search and selection process, from the number of records identified in the search to the number of studies included in the review, ideally using a flow diagram. | Figure 1 |
|  | 16b | Cite studies that might appear to meet the inclusion criteria, but which were excluded, and explain why they were excluded. | Figure 1 |
| Study characteristics | 17 | Cite each included study and present its characteristics. | Lines 194-204 |
| Risk of bias in studies | 18 | Present assessments of risk of bias for each included study. | Figure S1 |
| Results of individual studies | 19 | For all outcomes, present, for each study: (a) summary statistics for each group (where appropriate) and (b) an effect estimate and its precision (e.g. confidence/credible interval), ideally using structured tables or plots. | Table 1 |
| Results of syntheses | 20a | For each synthesis, briefly summarise the characteristics and risk of bias among contributing studies. | Lines 205-208 |
|  | 20b | Present results of all statistical syntheses conducted. If meta-analysis was done, present for each the summary estimate and its precision (e.g. confidence/credible interval) and measures of statistical heterogeneity. If comparing groups, describe the direction of the effect. | Lines 221-235; Table 3, 4, S3, S4 |
|  | 20c | Present results of all investigations of possible causes of heterogeneity among study results. | Not applicable |
|  | 20d | Present results of all sensitivity analyses conducted to assess the robustness of the synthesized results. | Table 4 |
| Reporting biases | 21 | Present assessments of risk of bias due to missing results (arising from reporting biases) for each synthesis assessed. | Not applicable |
| Certainty of evidence | 22 | Present assessments of certainty (or confidence) in the body of evidence for each outcome assessed. | Lines 236-237 |
| **DISCUSSION** | | |  |
| Discussion | 23a | Provide a general interpretation of the results in the context of other evidence. | Lines 245-265; lines 280-295 |
|  | 23b | Discuss any limitations of the evidence included in the review. | Lines 296-306 |
|  | 23c | Discuss any limitations of the review processes used. | Lines 296-306 |
|  | 23d | Discuss implications of the results for practice, policy, and future research. | Lines 309-321 |
| **OTHER INFORMATION** | | |  |
| Registration and protocol | 24a | Provide registration information for the review, including register name and registration number, or state that the review was not registered. | Lines 86-87 |
|  | 24b | Indicate where the review protocol can be accessed, or state that a protocol was not prepared. | Lines 86-87 |
|  | 24c | Describe and explain any amendments to information provided at registration or in the protocol. | Not applicable |
| Support | 25 | Describe sources of financial or non-financial support for the review, and the role of the funders or sponsors in the review. | Lines 333-335 |
| Competing interests | 26 | Declare any competing interests of review authors. | Lines 337-342 |
| Availability of data, code and other materials | 27 | Report which of the following are publicly available and where they can be found: template data collection forms; data extracted from included studies; data used for all analyses; analytic code; any other materials used in the review. | Lines 344-335 |

*From:*  Page MJ, McKenzie JE, Bossuyt PM, Boutron I, Hoffmann TC, Mulrow CD, et al. The PRISMA 2020 statement: an updated guideline for reporting systematic reviews. BMJ 2021;372:n71. doi: 10.1136/bmj.n71

For more information, visit: <http://www.prisma-statement.org/>

**Figure S1** – Methodological assessment


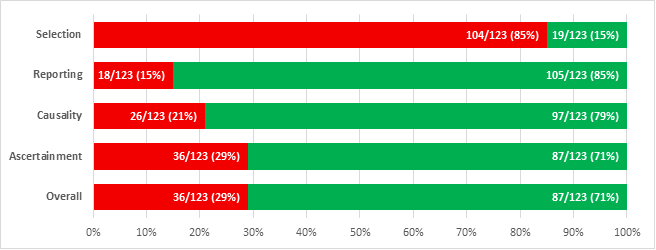


In **red** low methodological assessment, in **green** good methodological assessment, according to the methods section.

**Figure S2** - Geographical distribution of the individual cases reviewed, according to their numerosity by country of diagnosis


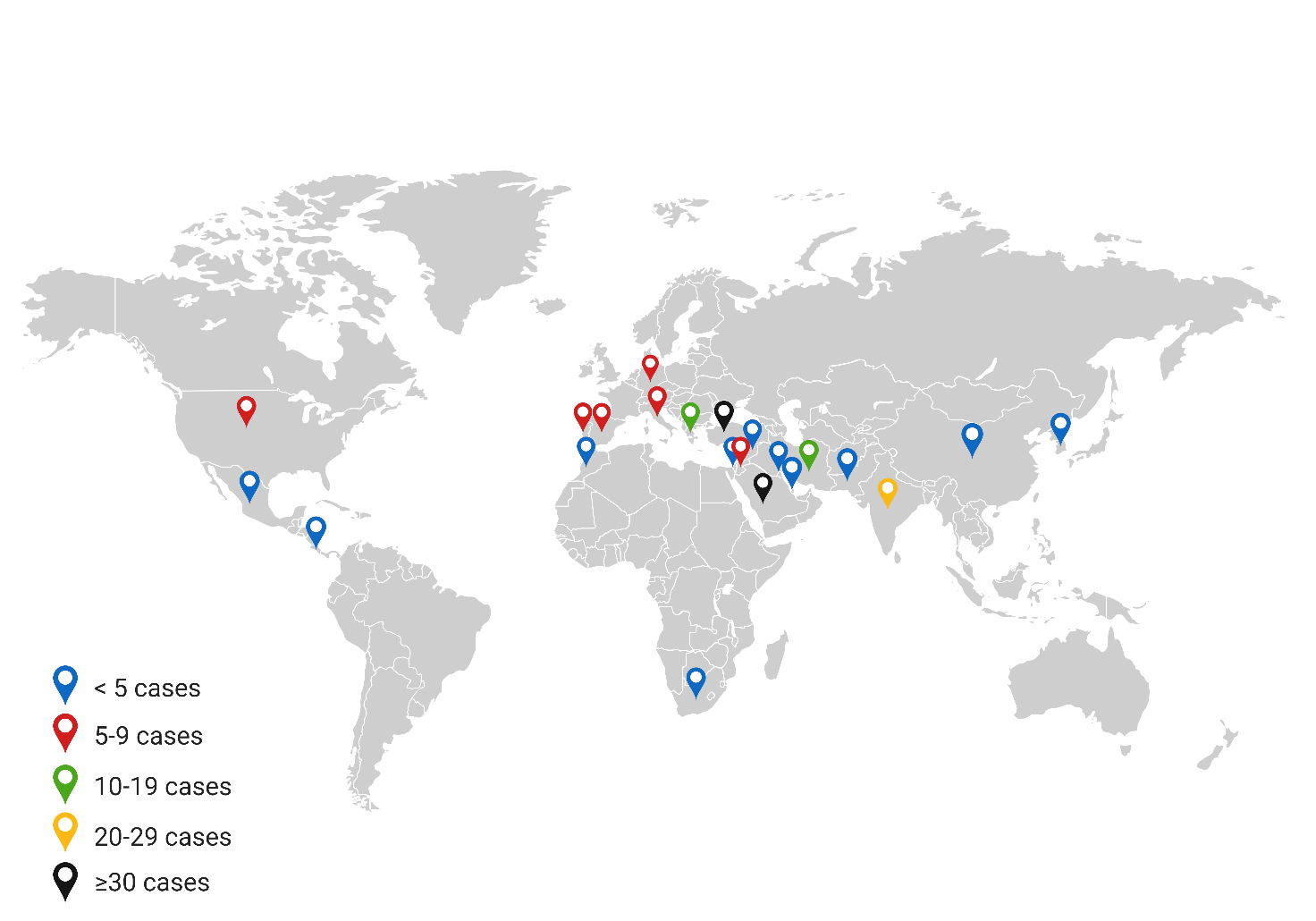

Supplement: Supplementary file 1 — Supplementary file1 (DOCX 175 KB) [file 10072_2024_7621_MOESM1_ESM.docx]
